# Irrational Use of Medicine in the Treatment of Presumptive Asthma Among Rural Primary Care Providers in Southwestern China

**DOI:** 10.3389/fphar.2022.767917

**Published:** 2022-02-15

**Authors:** Huidi Liu, Huibo Li, Dirk E. Teuwen, Sean Sylvia, Haonan Shi, Scott Rozelle, Hongmei Yi

**Affiliations:** ^1^ China Center for Agricultural Policy, School of Advanced Agricultural Sciences, Peking University, Beijing, China; ^2^ Department of Pharmacy, Peking University Third Hospital, Beijing, China; ^3^ Institute for Drug Evaluation, Peking University Health Science Center, Beijing, China; ^4^ Department of Neurology, Ghent University Hospital, Ghent, Belgium; ^5^ Department of Health Policy and Management and the Carolina Population Center, University of North Carolina at Chapel Hill, Chapel Hill, NC, United States; ^6^ Business Development Center, Red Cross Society of China, Beijing, China; ^7^ Center on China’s Economy and Institution, Stanford University, Stanford, CA, United States

**Keywords:** irrational use of medicine, asthma, primary care providers, clinical vignette, standardized patients

## Abstract

Poor knowledge, scarce resources, and lack of or misaligned incentives have been widely documented as drivers of the irrational use of medicine (IUM), which significantly challenges the efficiency of health systems across the globe. However, there is limited understanding of the influence of each factor on IUM. We used detailed data on provider treatment of presumptive asthma cases in rural China to assess the contributions of provider knowledge, resource constraints, and provider behavior on IUM. This study enrolled 370 village providers from southwest China. All providers responded to a clinical vignette to test their knowledge of how to treat presumptive asthma. Resource constraints (“capacity”) were defined as the availability of the prescribed medicines in vignette. To measure provider behavior (“performance”), a subset of providers (104 of 370) were randomly selected to receive unannounced visits by standardized patients (SPs) who performed of presumptive asthma symptoms described in the vignette. We found that, 54% (201/370) of providers provided the vignette-based patients with prescriptions. Moreover, 67% (70/104) provided prescriptions for the SPs. For the vignette, only 10% of the providers prescribed the correct medicines; 38% prescribed only unnecessary medicines (and did not provide correct medicine); 65% prescribed antibiotics (although antibiotics were not required); and 55% prescribed polypharmacy prescriptions (that is, they prescribed five or more different types of drugs). For the SP visits, the numbers were 12%, 51%, 63%, and 0%, respectively. The lower number of medicines in the SP visits was due, in part, to the injections’ not being allowed based on ethical considerations (in response to the vignette, however, 65% of providers prescribed injections). The difference between provider knowledge and capacity is insignificant, while a significant large gap exists between provider performance and knowledge/capacity (for 11 of 17 indicators). Our analysis indicated that capacity constraints play a minor role in driving IUM compared to provider performance in the treatment of asthma cases in rural China. If similar findings hold for other disease cases, this suggests that policies to reduce the IUM in rural China have largely been unsuccessful, and alternatives for improving aligning provider incentives with appropriate drug use should be explored.

## 1 Introduction

The rational use of medicine requires that “*patients receive medications appropriate to their clinical needs, in doses that meet their own individual requirements, for an adequate period of time, and at the lowest cost to them and their community*” (World Health Organization, 1985). A World Health Organization (WHO) report noted that less than 40% of primary care patients in public facilities and 30% in the private sector of developing and transitional countries are treated following standard treatment guidelines (Holloway and Van Dijk, 2011). The irrational use of medicine (IUM), in particular, IUM by providers, is a major challenge that affects healthcare systems worldwide. Examples of provider IUM include polypharmacy; inappropriate use of antibiotics for non-bacterial infections; overuse of injections when oral formulations would be more appropriate; and failure to prescribe in accordance with clinical guidelines (De Vries et al., 1994; Hogerzeil et al., 2001; World Health Organization, 2021). Harmful consequences of IUM include adverse events, increasing antimicrobial resistance, and the spread of blood-borne infections, all of which can result in increased morbidity and mortality (Holloway and Van Dijk, 2011). IUM is also thought to be a significant source of unnecessary medical expenditures. Medications account for 70–75% of total health expenditures in low- and middle-income countries (LMICs), and WHO has estimated that 50–70% of these medications are not needed and constitute IUM (World Health Organization, 2008; Ofori-Asenso and Agyeman, 2016).

Existing studies suggest three main reasons for IUM in LMICs. First, inadequate knowledge and poor training of health professionals have been shown to be highly correlated with a high prevalence of IUM in LMICs (World Health Organization, 2002; Das et al., 2012; Mao et al., 2015; Mohamadloo et al., 2017; Machowska and Lundborg, 2019). Second, there is evidence that structural factors related to limited health system resources (*e.g.*, the unavailability of medical equipment, unrestricted availability of medications, high caseloads) are negatively associated with appropriate prescribing practices and the ability of providers to apply their knowledge (Das and Hammer, 2014; Giorgio et al., 2020). Third, studies of IUM in different settings have found that institutional features, including financial incentives, promotion of medications, and system-wide legislations as well as sociocultural factors and political issues, to affect the behavior of providers (World Health Organization, 2002; Holloway and Van Dijk, 2011; Mao et al., 2015; Mohamadloo et al., 2017). These institutional and other features also can amplify knowledge deficits of both providers and patients (Mao et al., 2013).

This study aimed to analyze the relative contribution of these three factors to IUM in village clinics in rural China. We used data from a survey of providers in village clinics—the most common first point of patients (Babiarz et al., 2010)—in a southwestern province to measure provider IUM in the treatment of a presumptive case of asthma. We decomposed the total scale of IUM into the *know* gap that is attributable to deficits in provider knowledge to achieve the expected level of appropriate prescription; the *know-can* gap, which is measured by the difference between provider knowledge and what they can do, given available resources; and the *can-do* gap, which is measured by the difference between what they can do and what they actually do in practice (Lange et al., 2014; Mohanan et al., 2015; Ibnat et al., 2019). The three gaps are related to the three main factors, as described above, associated with IUM in LMICs. Specifically, the *know* gap is about provider education and training; the *know-can* gap is related to structural constraints; and the *can-do* gap involves factors that influence the efforts of providers (Das and Hammer, 2014).

## 2 Methods

A survey of village providers in a random sample of 370 villages in Yunnan Province (southwestern China) was used to collect information on provider knowledge (*know*), capacity (*can*), and performance (*do*) in the case of the provider’s ability to prescribe medicines properly and completely for the treatment of a presumptive case of asthma. In the survey, a standardized clinical vignette was used to assess the *know* part of the study; data on clinic resources, to measure the *can* part of the study; and unannounced visits by standardized patients (SPs), to ascertain the *do* part of the study. Asthma was chosen as the disease, given its high burden in China, with more than 45 million individuals affected, coupled with the fact that previous studies have documented high rates of IUM in the treatment of the disease (Osaretin et al., 2013; GBD 2015 Chronic Respiratory Disease Collaborators, 2017; Ding and Small, 2018).

### 2.1 Setting

Data were collected from rural village providers in three prefectures of Yunnan Province between July 2017 and January 2018. The *per capita* gross domestic product in Yunnan Province was 34,221 RMB ($5,068 US^1^) in 2017, ranking it as the second poorest province in China. The rural population in 30 counties of the three sample prefectures was 6.5 million, out of a total rural population of 46 million in the province (Yunnan Bureau of Statistics of China, 2017).

China’s rural primary care system is comprised of three tiers of providers—county hospitals, township health centers, and village clinics. The village clinic is the front line of the health system and is designed to provide outpatient care, the sale and dispensing of medications for common clinical conditions, and the provision of public health services. Village clinics typically function as independent for-profit entities and, at least in the past, earned part of their revenue from the sale of medications to their patients. As one of five key areas of the comprehensive health reform, the Chinese central government introduced the Zero-Markup Drug Policy (ZMDP) of the drugs on the Essential Drug List (EDL) in 2009 and has updated the EDL every 3 years since then (China, 2009; The Central Committee and the State Council of the Communist Party of China, 2009). In addition, provincial governments can add drugs to the list based on local needs. The policy intended to ensure safety in the use of medications and controls for drug-related expenditures by decoupling the compensation of healthcare providers, including government-funded village providers, from drug prescriptions and sales (China, 2009). After the comprehensive health system reform, the central government suggested a fixed quota of subsidies, which is referred to payments to village cadres, to compensate village providers, including subsidizing their loss due to implementation of the ZMDP (General Office of State Council of China, 2013). There is anecdotal evidence, however, that indicates that, after the introduction of the EDL, the drugs that village providers used to prescribe, and patients needed, were not available in village clinics (Guan et al., 2018).

### 2.2 Study Population

**FIGURE 1:**
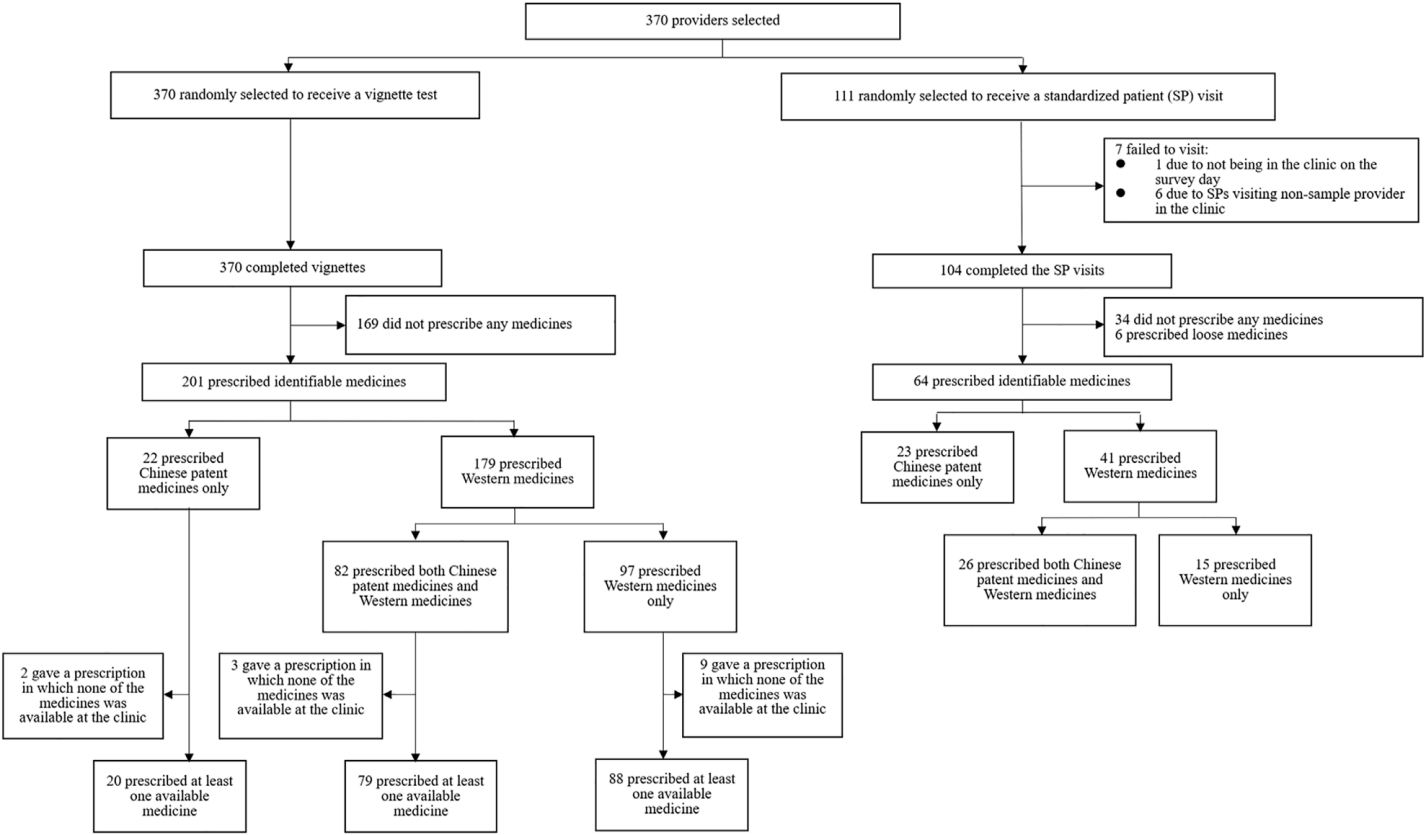
Strobe flowchart.

The full sample of the study comprised 370 village providers (Yi and Sylvia, 2017). To choose the sample, first, the research team randomly selected a total of 370 village clinics out of a comprehensive list of the 1,320 village clinics in the 10-county study area. Second, in each village clinic, the provider who was in charge of prescribing Western drugs and who assumed the main responsibility for outpatient care was selected. If more than one such provider was present, one was randomly selected. All 370 providers completed the provider survey instrument and clinic survey and participated in responding to the standardized asthma clinical vignette. Third, from the full sample, the research team randomly selected 111 providers who were assigned to be part of the study in which a standardized patient (SP) with presumptive asthma visited the provider’s clinic (Figure 1).

### 2.3 Data Collection

The data for this study was collected through three surveys, as described below.

#### 2.3.1 Provider and Clinic Surveys

The first survey, conducted in July 2017, was used to collect detailed information on the full sample of village providers and their clinics. The provider survey contained items in regard to the respondents’ age, gender, highest level of education attained, medical certification, training in area of the diagnosis and treatment of asthma, training in the use of antibiotics, familiarity with the term *clinical pathway*,^2^ and the share of their income that was earned from their work in the clinic.

The clinic facility survey was used to collect information on the number of providers in the clinic, number of patient visits during the previous month, composition and size of the stock of Western medicines and Chinese patent medicines (CPMs), availability of medical instruments that may be used for the diagnosis of asthma, availability of intravenous drip equipment, availability of intra-muscular injection tools/equipment, whether the clinician kept medical records, any incentives from upper levels tied to prescription assessment, and implementation of the EDL and ZMDP. A translated English version of the provider survey form and clinic facility survey form are presented in Supplementary Appendices SA, SB.

#### 2.3.2 Unannounced Standardized Patient Visit

The second round of the survey process, in December 2017, consisted of unannounced SP visits to selected providers. The unannounced SP method is considered the gold standard for measuring actual clinical practice in the real world (Das and Hammer, 2014; Das et al., 2016). In comparison with other methods of measuring healthcare quality, it has the advantage of enabling both case and patient mix (Das and Hammer, 2014). An SP script that presented a case of presumptive asthma, previously used in a study in India, was translated and adapted to the context in China with the assistance of respiratory specialists from the Peking Union Medical College Hospital and from People’s Hospital, Peking University (Das et al., 2016). The SP script includes disease signs and symptoms, medical history, and patient background (Supplementary Appendix SC1).

To implement the SP protocol, five around 25-year-old women were recruited from local communities as SPs and trained intensively for approximately 2 weeks to enable them to make a consistent presentation of the disease case to providers. Before the training, the SPs had been given a physical examination in the university hospital to ensure they were healthy and had no physiological symptoms that might affect the diagnosis of village providers. The training focused on standardizing disease case presentations across SPs and on safety measures for SPs to avoid invasive procedures during clinic visits. For the latter, SPs were provided with standardized phrasing to refuse invasive procedures (Supplementary Appendix SC2).

The intention of the SP protocol was to make all visits as consistent as possible. The SPs were randomly assigned to providers and followed the normal procedures for any walk-in patient. Upon being presented to the provider, SPs made an opening statement of the primary symptoms of the case: “Doctor, I have a shortness of breath; I am wheezing.” The SPs would respond to all questions posed by the provider, following a standardized script, purchase all medicines prescribed (which are sold by the providers), and pay the provider any fee. As noted, all of the SPs were trained to reject invasive procedures during clinic visits.

Following each visit, each SP was debriefed using a structured questionnaire, and SPs’ responses were confirmed against a recording of the interaction taken, using a concealed recording device. The structured questionnaire detailed the provider’s questions (which were compared to an approved checklist of appropriate/necessary questions), diagnostic examinations and tests requested, the stated diagnosis, the treatment prescribed (drugs or advice), and patient referral(s) (Supplementary Appendix SC3). In terms of drugs, the information included the name of the medicine, dosage form, nature/form of administration, and the dosage regimen (the time between doses and the amount of a medicine to be given at each specific time).

#### 2.3.3 Standardized Clinical Vignettes

In January 2018, enumerators returned to sample clinics to administer a standardized clinical vignette to the sample providers. The clinical vignette was designed to present the same asthma case as was presented by the SPs, except for two changes. One was that providers were aware that they were being evaluated; (Das and Hammer, 2014) the other was that intrusive procedures (*e.g.*, injections) were allowed.

A total of 32 enumerators (2 per team, 16 teams) were trained for 7 days to present the standardized clinical vignette to the sample providers. One enumerator in a team assumed the role of a “mock patient”; the other assumed the role of “facilitator.” The facilitator read the instructions to the provider, documented the interaction, and provided additional information that the patient might not know but would be helpful to the provider’s diagnosis if the provider actively solicited it, *e.g*., the results of tests or examinations. At the start of the clinical vignettes, providers were told to consult with the “mock patient” as they would a patient in their clinic but to assume that they had access to any diagnostic equipment and therapeutics they required.

During the vignette interactions with the sample providers, the enumerators documented the same information as was recorded in the structured questionnaire of SP visits. Although the team collected information about the name of the medicines and routes of administration of each drug prescribed, only detailed information was documented in regard to the dosage regimen for drugs that were available in the clinic. For those that providers prescribed, but were not available at clinic, the vignette teams did not collect such information. Data are provided in Supplementary Appendix SD.

### 2.4 Outcomes

#### 2.4.1 Measurement of IUM

**FIGURE 2:**
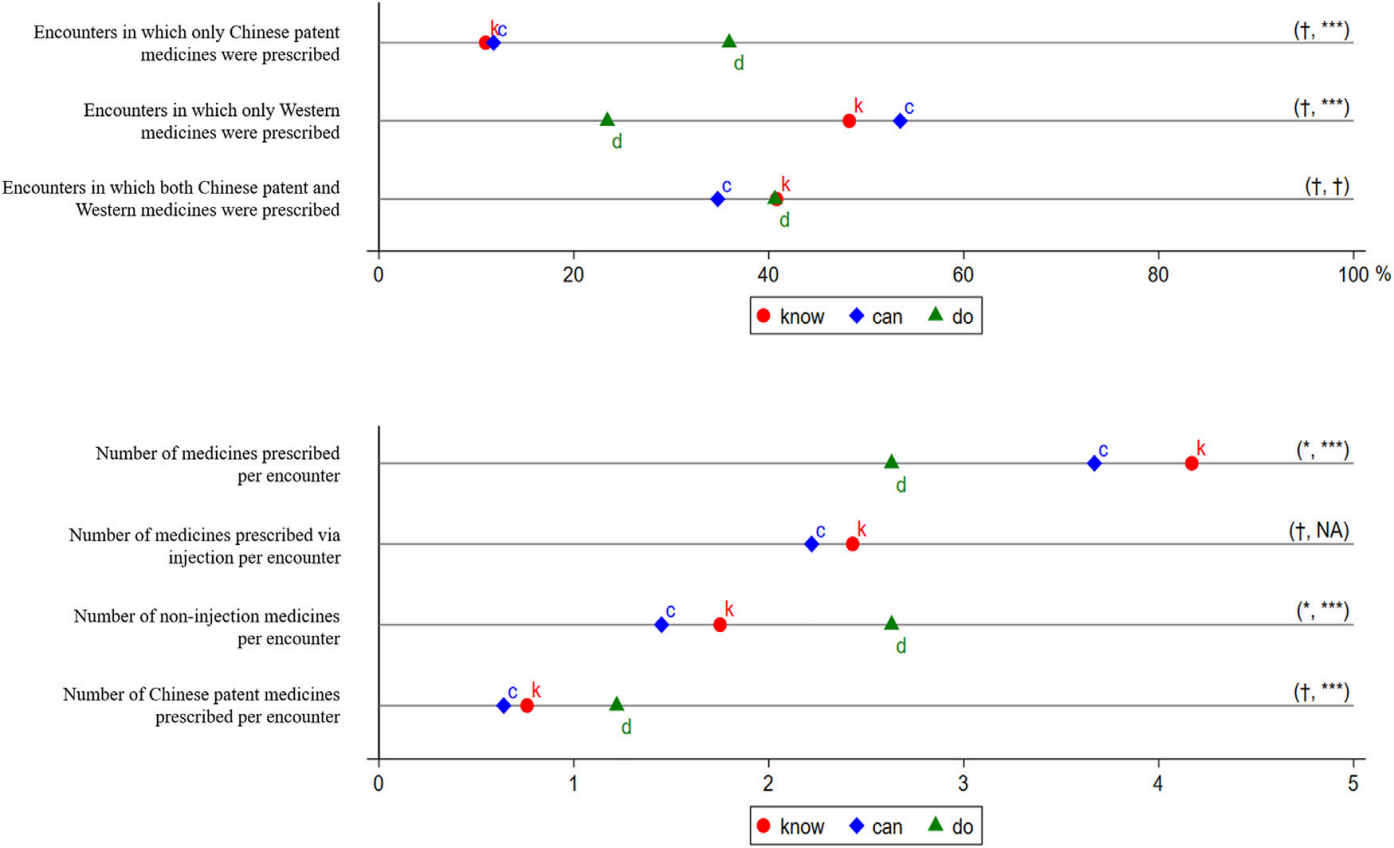
Prescriptions in the Treatment of Presumptive Asthma. *k*, *c*, and *d* represent *know*, *can*, and *do*, respectively. The *know* gap is not measured because no well-recognized target performance is available. The distance between *k* and *c* is the *know-can* gap, and the distance between *c* and *d* is the *can-do* gap. We use t-tests to examine whether the gaps are statistically significant and report the significance of the *know-can* gap and the *can-do* gap in parentheses. †*p* > 0.05, *p < 0.05, **p < 0.01, ***p < 0.001. We did not consider Chinese patent medicines in measuring the outcomes. Injection included intravenous drip and intramuscular injection.

The survey data were used to produce three categories of outcome variables. Specifically, information on drug prescriptions was used to create a primary set of measures of IUM. From this information, we produced variables that included the number of providers who prescribe medicines, number of different drugs, origin of each (Western *vs*. CPMs), and route of administration of the medicines (Figure 2).

**FIGURE 3:**
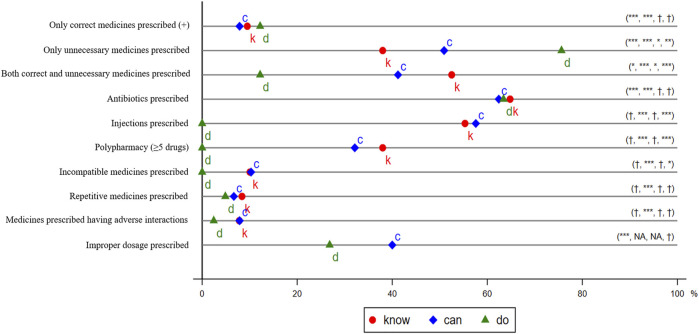
Irrational Use of Medicine in the Treatment of Presumptive Asthma. *k*, *c*, and *d* represent *know*, *can*, and *do*, respectively. The distance between 0 and *d* and the distance between 0 and *k* is the *total* gap and the *know* gap, respectively, except that the *total* gap and *know* gap of *only correct medicines prescribed* is the distance between 100% and *d* or *k*. The distance between *k* and *c* is the *know-can* gap, and the distance between *c* and *d* is the *can-do* gap. We use t-tests to examine whether the gaps are statistically significant and report the significance of *total* gap, *know* gap, *know-can* gap, and *can-do* gap in parentheses. †*p* > 0.05, *p < 0.05, **p < 0.01, ***p < 0.001. We did not consider Chinese patent medicines in measuring the outcomes. Injection included intravenous drip and intramuscular injection.

The second category outcomes were whether prescriptions followed the Chinese National Practice Guidelines to treat asthma (Asthma Workgroup of Chinese Thoracic Society and Chinese Society of General Practitioners, 2013; Asthma Workgroup of Chinese Thoracic Society, 2016). Each Western medicine on the prescription list was classified as necessary (or correct), unnecessary, or potentially harmful (Supplementary Appendix SE). Based on the combination of drugs in a prescription, seven indicators were reported: only correct medicines prescribed; only unnecessary medicines prescribed; only potentially harmful medicines prescribed; both correct and unnecessary medicines prescribed; both correct and potentially harmful medicines prescribed; both unnecessary and potentially harmful medicines prescribed; and correct, unnecessary, and potentially harmful medicines prescribed together (Figure 3).

A third category of outcome variables was defined according to Chinese Pharmacopoeia and Standards for Prescription Examination in Medical Institutions (Figure 3) (National Health Commission of the People’s Republic of China, 2018; Commission, 2020). These indicators are widely used in recent literature on rationing use of medicines (World Health Organization, 1993; World Health Organization, 2002; Dong et al., 2010; Dhamija et al., 2013; Ventola, 2015; Ofori-Asenso and Agyeman, 2016; Rajalingam et al., 2016; Fujita et al., 2018). Specifically, we assessed whether any antibiotics were prescribed, any injections were prescribed, five or more drugs were prescribed (which we define as *polypharmacy*), medicines were prescribed without indication, incompatible medicines were prescribed, repetitive medicines were prescribed, medicines prescribed had adverse interactions, and improper dosages were prescribed. The last outcome could be assessed only for the prescribed medicines that were available in the clinics.

Two pharmacists from the Peking University Health Science Center independently evaluated and scored the prescriptions using predetermined protocols. When the scores were different, a third pharmacist would engage in discussion with them and reach a consensus. For an international comparison, CPMs were excluded in the assessment of the last two types of outcomes.

#### 2.4.2 Measurement of *Know*, *Can*, *Do*, and Three Gaps

First, we measured the provider’s *know*, *can*, and *do* for each outcome. Specifically, we used the provider’s actual performance in the SP visits to measure the provider’s *do*. The provider’s performance in the clinical vignette was a measure of the provider’s *know* (Das and Hammer, 2014). The provider’s *can* was defined by the availability of the prescribed medicines during the clinical vignette in the dispensing pharmacy of the village clinic (Ibnat et al., 2019).

Then, we calculated the total gap, using the difference between the average of the providers’ actual performance (*do*) and the target performance. We defined target performance as perfect adherence to prescription guidelines. We do not report the total gap for the first type of outcome because there were no available well-recognized guidelines for this outcome. In terms of the second and the third categories of outcomes, the target performance was assumed to be zero, except that it was 100% for the outcome of whether only correct medicines were prescribed.

Finally, we decomposed the total gap into three gaps. The first gap was the *know* gap, which was the difference between the average of the provider’s *know* and the target performance. The *know* gap was defined as the part that could be attributed to the provider’s knowledge deficiency in the total gap. The second gap was the *know-can* gap, or difference between the average of the provider’s *can* and the average of the provider’s *know*. An insignificant *know-can* gap indicates that the availability of drugs did not affect the rational use of medicine (RUM). The third gap was *can-do*, defined as the difference between the average of the provider’s *can* and the average of the provider’s *do*. The *can-do* gap reflects the part of the provider’s efforts, excluding knowledge deficiency and resource constraints from the total gap.

### 2.5 Statistical Methods

Statistical analyses were conducted using Stata 15.1. The means and standard deviations (SDs) of continuous variables, numbers, and percentages of binary variables were reported. In addition, *t*-tests of the equality of means between *know* and *target performance* (or the *know* gap) and between *know* and *can* (or the *know-can* gap) were conducted for paired data; *t*-tests of the equality of means between *can* and *do* (or the *can-do* gap) were conducted for unpaired data. Whether a 95% confidence interval (CI) of the difference in the mean of outcomes contains 0 was used to assess the statistical significance of the gaps.

### 2.6 Ethics and Informed Consent

Full ethical approval for this survey was obtained from the Peking University Institutional Review Board on April 26, 2017 (IRB00001052-17033). The Board approved the verbal consent procedure, and verbal consent was obtained from local health departments and participants at the start of the survey. We collected verbal consent for the following reasons. The research presented minimal risk of harm to subjects and involved no procedures for which written consent was normally required outside of the research context, and verbal consents are more culturally acceptable than are written consents in the region.

## 3 Results

### 3.1 Characteristics of Providers and Clinics

The average age of the 370 providers was 46 years, and 64% are male. In addition, 68% graduated senior high school, 26% completed junior college or a higher level of education, and 6% completed junior high school or had a lower level education. Only 8% were qualified medical practitioners or qualified associate medical practitioners, and 18% reported they were familiar with the term *clinical pathway*.

No provider received training in regard to asthma in the previous 2 years, and 54% received training in use of antibiotics. In terms of income, 95% came from working in the clinic.

Each village clinic had, on average, two providers. The number of patient visits in the previous month was 601 (SD = 1,662) with the median 303.^3^ Almost all clinics implemented the ZMDP in regard to essential drugs. At the clinic, 95 brands of Western medicines and 42 brands of CPMs were available. Medical instruments and services for chest auscultation, assessment of heart rate/pulse/blood pressure, and percussion added to chest examination were available in almost all clinics, whereas those for a pulmonary ventilation test, bronchodilator test, chest X-ray examination, or blood routine test were seldom or not available. Almost all clinics provided an intravenous drip or intramuscular injection, 84% kept medical records, and 81% reported incentives tied to prescription assessment.

### 3.1 Prescription in the Treatment of Presumptive Asthma

#### 3.2.1 Providers’ Performance in SP Visits

As illustrated in Figure 1, 67% (70/104) of providers prescribed in SP visits, and six (6%) provided loose medicines (medicines taken from original packaging and did not provide a written or oral prescription) in the SP visits. Because the exact name of these medicines and related information was undeterminable, the six providers with loose medicines were excluded from the remainder of the analysis.

The descriptive statistics of prescriptions in the treatment of presumptive asthma are seen in Figure 2. For the composition of each prescription by the origin of medicines, we found that, in SP visits, 36% (23/64) of the prescriptions included CPMs alone; 23% (15/64), Western medicine(s) alone; and 41% (26/64), CPMs and Western medicine(s) combined (Figure 2, Panel A). Because injections, as an intrusive treatment, were not allowed in SP visits, only non-injectable medicines were prescribed. The number of medicines per encounter was 2.63 (±1.28), of which providers prescribed 1.22 (±0.95) CPMs per encounter in SP visits on average.

#### 3.2.2 Providers’ Know-Can Gap, and Can-Do Gap in Prescriptions

Although nearly two-thirds of providers prescribed in SP visits, fewer providers prescribed in the case of the vignette. Specifically, 54% (201/370) prescribed in a vignette (Figure 1), and this share fell to 51% (187/370) due to medicine unavailability. Even among 104 providers who completed both vignettes and SPs, 17 (16.3%) prescribed neither in a vignette nor an SP visit, 39 (37.5%) prescribed in both a vignette and SP visit, 17 (16.3%) prescribed only in a vignette, and 31 (29.8%) prescribed only in an SP visit. There were no significant differences in most of the characteristics between those who prescribed only in a vignette and those who prescribed only in an SP visit (Supplementary Appendix Table S1). An unpaired *t*-test indicated that the *know-can* gap was not statistically significant (*p* > 0.05) (Supplementary Appendix Table S2). The *can-do* gap, however, was statistically significant (*p* < 0.01) and suggests that providers were more likely to prescribe in an SP visit than in a vignette, without or with consideration of medicine unavailability, respectively, by 13 percentage points (by 24%) and 17 percentage points (by 33%).

We also found that providers were more likely to prescribe CPMs in an SP visit than in a vignette (Figure 2, Panel A). Specifically, in a vignette, 11% (22/201) of the prescriptions included CPMs alone; 48% (97/201), Western medicine(s) alone; and 41% (82/201), CPMs and Western medicine(s) combined. Even taking medicine unavailability into account, 12% (22/187) of the prescriptions included CPMs alone; 53% (100/187), Western medicine(s) alone; and 35% (65/187), CPMs and Western medicine(s) combined. Although the *know-can* gaps of prescribing CPMs alone or prescribing Western medicine(s) alone were not statistically significant (*p* > 0.05), the *can-do* gaps were statistically significant (*p* < 0.001). This shows that providers were more likely to prescribe CPMs only in an SP visit by 24 percentage points (by 200%), and less likely to prescribe Western medicines only by 30 percentage points (by 56%) than in a vignette with consideration of medicine unavailability. Neither the *know-can* gap nor the *can-do* gap of prescribing a combination of CPMs and Western medicines were statistically significant (*p* > 0.05).

The results also show that providers prescribed more medicines via other administration routes in an SP visit, where injections were not allowed, than they did in a vignette (Figure 2, Panel B). In vignettes, providers prescribed 4.17 (±2.47) medicines, of which 2.43 (±2.73) were prescribed via injection. Even after excluding unavailable medicines, providers prescribed 3.67 (±2.39) medicines, of which 2.22 (±2.53) were prescribed via injection. The number of non-injection medicines per encounter declined slightly, from 1.75 (±1.36) to 1.45 (±1.25), due to medicine unavailability (*p* < 0.05). Because injections were not allowed in SP visits, no medicines via injection were prescribed (even though they were deemed necessary by some providers). The number of non-injection medicines per encounter increased to 2.63 (±1.28) in SP visits, an increase of 81% (*p* < 0.001) in comparison with a vignette with consideration of medicine availability. In addition, providers prescribed fewer CPMs in a vignette without or with consideration of medicine unavailability, respectively, 0.76 ± 0.89 and 0.64 ± 0.80, than that in an SP visit (1.22 ± 0.95).

### 3.3 Compliance of Drug Use to Guidelines in the Treatment of Presumptive Asthma

#### 3.3.1 Providers’ Performance in SP Visits and the Total Gap

In Figure 3, we first present the data on whether the use of medicines followed the guidelines of the treatment of presumptive asthma. More details can be found in Supplementary Appendix Table S3. In SP visits, 64% (41/64) of providers prescribed at least one Western medicine. Among them, 12% (5/41) prescribed only correct medicines; 76% (31/41), only unnecessary medicines; 12% (5/41), both unnecessary medicines and correct medicines; and none prescribed harmful medicines. The total gap for correct prescription was significant (*p* < 0.001), as large as 88 percentage points (the target performance is 100%), and the total gap of unnecessary prescription was −76 percentage points (the target performance is 0) and statistically significant (*p* < 0.001).

Figure 3 also presents the data for the assessment of seven frequently cited indicators of IUM. For each of these indicators, providers performed as follows in the SP visits: antibiotics prescriptions (63%), injection prescriptions (0%), polypharmacy (0%), incompatible medicine prescriptions (0%), repetitive medicine prescriptions (5%), prescriptions with adverse interaction (2%), and improper dosage prescribed (27%). A *t*-test indicated that, except for prescription of antibiotics (*p* < 0.001) and improper dosage (*p* < 0.001), the total gaps of the other five variables are statistically insignificant.

#### 3.3.2 Decomposition of the Total Gap: Know Gap, Know-Can Gap, and Can-Do Gap

We decomposed the total gap of each outcome into three gaps: *know* gap, *know-can* gap, and *can-do* gap. We found that the total gap in correct prescription was driven mainly by the *know* gap. Specifically, in vignettes, around 10% (17/179) prescribed only correct medicines. The *know* gap of prescribing correctly was 90 percentage points and statistically significant (*p* < 0.001). Nevertheless, neither the *know-can* gap nor the can-do gap for prescribing correctly was statistically significant (*p* < 0.05).

In contrast, the total gap for prescribing unnecessarily was found to be attributable to all three gaps. In vignettes, 38% (68/179) of providers prescribed only unnecessary medicines, and the knowledge gap is statistically significant (*p* < 0.001). This share increases to 51% (84/165) when we excluded unavailable medicines. The *know-can* gap of prescribing unnecessarily was statistically significant (*p* < 0.05). Further, the statistically significant *can-do* gap (*p* < 0.01) suggests that providers were more likely to prescribe only unnecessary medicines in an SP visit than in a vignette by 25 percentage points (by 49%), even in consideration of medicine availability. We also found that the total gap for prescribing both correct and unnecessary medicines was due to all three gaps simultaneously. No gaps were found in the prescription of drugs that might be harmful to the treatment of presumptive asthma.

In terms of the third categorical outcome, we found that the total gap for antibiotics prescription, prescription of repetitive medicines, and prescription with adverse interacting medicines were driven mainly by the knowledge gap. The *know-can* and *can-do* gaps were statistically insignificant (*p* > 0.05). Although the total gaps of injection prescription, polypharmacy, and prescription of incompatible medicines were zero, this appears due to the *can-do* gap offsetting the knowledge gap. Providers’ performance in the three indicators are poor in a vignette, regardless of medicine availability, and they performed perfectly in SP visits because injections were prohibited. We did not report the percentage of prescriptions with improper dosages in the vignette because we collected information only about the dosage regimen for available drugs in the clinics. An analysis of a subsample of 104 providers who completed both the vignette and an SP indicated consistent results (Supplementary Appendix Tables S4, S5).

## 4 Discussion

This assessment of providers’ knowledge, capacity, and performance in the use of medicines in the treatment of presumptive asthma was conducted in a rural setting, with 370 rural village providers in three prefectures in Yunnan Province. Three categorical outcomes (description on prescription, compliance with the clinical guideline to treat asthma, and widely used indicators in recent literature on rational use of medicine) consisting, respectively, of nine, seven, and seven indicators were assessed. The total gap between actual performance and target performance was decomposed into three gaps: *know* gap, *know-can* gap and *can-do* gap.

Of the second and third categorical outcomes (14 indicators) with well-defined target performance, the total gap of five indicators (only correct medicines prescribed, only unnecessary medicines prescribed, both correct and unnecessary medicines prescribed, antibiotics prescribed, improper dosage prescribed) was statistically significant. Eleven indicators were characterized by insignificant total gaps.

When we decomposed the total gap into three gaps, we found that knowledge of all indicators is statistically significant, whereas the *know-can* gap was not statistically significant in most cases. Of the assessed *can-do* gap of 17 indicators, 11 were statistically significant. Of these, six indicators were positive, indicating that providers performed better in an SP visit than they did in a vignette, and five were negative, indicating providers performed worse in SP visits than they did in a vignette.

In the clinical vignettes, the number of medicines prescribed per encounter was found to be higher than previously reported in another study in China and Vietnam as well as international standards (4.17 versus 2.19) (Mao et al., 2015). More than half of the medicines (58%; 2.43/4.17) were prescribed via injections that were unnecessary for the treatment of the case. In particular, although unnecessary, antibiotics were the most frequently prescribed medicine in our study. Prescriptions of antibiotics in this study, at 64%, revealed a much higher rate when compared to that of the WHO/International Network of Rational Use of Drugs (INRUD) (30% of prescriptions in general), (Joncheere, 2002) asthma, or angina SP visits to informal health providers in India (33%), (Das et al., 2016) or adult respiratory cases in the United States (23%) (Shapiro et al., 2014). We also noted, however, that this number is similar to that of asthma or angina SP visits to healthcare providers in primary health centers in India (64%) (Das et al., 2016) and children’s asthma cases in China (50–100%) (Chen et al., 2013; Wu et al., 2020). The univariate analysis revealed an insignificant correlation (*p* > 0.05) between a prescription of antibiotics and provider characteristics, including whether the provider received training in antibiotics. This suggests that the training might be ineffective to reduce the knowledge gap (Supplementary Appendix Table S6).

Providers were more likely to prescribe, in particular, CPMs in SP visits than in vignettes. On the one hand, we found that some providers (17/104; 16%) changed from prescribing in a vignette to not prescribing in an SP visit. According to earlier research, after the EDL and ZMDP, many drugs that doctors used to prescribe became unavailable (Wang et al., 2015; Yang et al., 2015). We excluded this possibility, however, because, of the 17 prescriptions, there are 16 in which at least one drug in the prescription was immediately available at the clinics. Another possibility is that providers might change to not prescribing when their diagnostic capacity is constrained by unavailable examination and testing tools in an SP visit (see limitations of this study). Further, 30% (31/104) providers changed to prescribing in SP visits from not prescribing in clinical vignettes. When we looked at the prescription by the origin of medicines, we found that the statistically significant *can-do* gap indicates higher incentives for providers to prescribe, in particular, CPMs and unnecessary medicines. There could be two explanations for the two gaps. One is that providers could earn more from selling CPMs instead of Western medicines (Zhang et al., 2015; Cai et al., 2020). The other is that providers might believe that CPMs would be “safer” than Western medicines when fewer adverse drug reaction cases were reported for CPMs (although this is not necessarily true), (Mossialos et al., 2016; National Medical Products Administration, 2020) particularly when they could not diagnose the case.

The number of encounters in which only unnecessary drugs were prescribed in an SP visit is almost twice that in a clinical vignette without (76 vs. 38%) and with (76 vs. 51%) consideration of drug availability. Inappropriate incentives for drug dispensing has been widely cited as a factor that encourages providers to prescribe more unnecessary drugs than they should (World Health Organization, 2002; Holloway and Van Dijk, 2011; Mao et al., 2015; Mohamadloo et al., 2017). Another explanation is that providers were more likely to prescribe unnecessary drugs in an SP visit because there were more uncertainties in the diagnosis due to unavailability of medical equipment for examinations and tests.

We found that, with fewer medicines prescribed per encounter in an SP visit, providers reduced the proportion of polypharmacy, medicines prescribed without indication, and incompatible medicines prescribed. The reduced number of medicines per encounter is very likely due to prohibition of injections in SP visits (due to ethical considerations). An injection prescription is a very common practice in China (Li et al., 2012; Yang et al., 2012; Mao et al., 2013; Mao et al., 2015). The medicines via injection accounted for 58% (2.43/4.17) in each encounter in clinical vignettes.

This study has three primary limitations. The first limitation concerns the measurement of capacity. The capacity of providers was defined by the availability of medicines, which may bias the capacity in uncertain ways. On the one hand, it might underestimate the capacity as, for example, providers may use a tele-medication system to improve their capacity for RUM (although anecdotally rare). On the other hand, it might overestimate providers’ capacity for RUM, as we did not take providers’ diagnostic capacity into account. As shown in Supplementary Appendix Table S7, RUM is related to the correctness of a diagnosis. Specifically, in a vignette, providers who gave a correct or partially correct diagnosis would be less likely to prescribe CPMs alone (1 vs. 16%, *p* < 0.01), to prescribe CPMs and Western medicines together (23 vs. 50%, *p* < 0.001), unnecessary medicines alone (13 vs. 53%, *p* < 0.001), antibiotics (51 vs. 73%, *p* < 0.01), and fewer CPMs per encounter (0.29 vs. 1.00, *p* < 0.001) than would whose who gave the wrong diagnosis; whereas they were more likely to prescribe Western medicines alone (75 vs. 34%, *p* < 0.001) and medicines that have adverse interactions (15 vs. 4%, *p* < 0.01) than would those who gave the wrong diagnosis. Further, we found that 25% of providers requested the results of a chest X-ray examination, but only one clinic had the needed instrument (*p* < 0.001; Supplementary Appendix Table S8).

The second limitation of this study is that some predetermined, inimitable and essential symptoms could be detected by the examinations and then might bias the diagnosis and results. In our study, there were seven recommended examinations for the diagnosis (Supplementary Appendix Table S10). For three of them, village providers would receive a similar result compared to the vignette if they implemented these examinations. Meanwhile, there were four examinations which would detect a different physiological symptom in the SP consultation when compared to the vignette consultation as SP were healthy and did not present such symptoms nor mimick them. Nevertheless, of the four examinations, two were prohibited to implement in SP visits because they were considered intrusive examination. Although percussion was allowed, no village providers performed. In SP visits, 20 (19%) village providers performed chest auscultation. Comparing those who performed chest auscultation in SP visits and those who did in vignette, we found that the rates of correct diagnosis were statistically insignificant (44 vs. 15%, *p >* 0.10 with 1,000 replications). In summary, we could reasonably conclude that the bias caused by this limitation would be negligible in this study but should be addressed in the future.

The third limitation of this study is that we excluded CPMs in measuring the second and third type of outcomes for IUM for the purpose of an international comparison. Nonetheless, we also provide the results for full prescriptions, including CPMs (Supplementary Appendix Table S9). These results are consistent with our findings, except for a higher incidence of improper dosage when CPMs are included (49 vs. 35%, *p* < 0.01).

## 5 Conclusion

Since 2009, China has launched major reform initiatives to improve the health sector performance for affordable, equitable, and effective health care for all by 2020. Primary care has been a central organizing paradigm for key health system functions policy reforms. This study found that, in general, resource constraints are not a major factor that drives IUM in rural China. More investments should be focused on improving providers’ knowledge and aligning provider efforts. In addition, more research is needed to build the link between policy and the narrowing of gaps. In this regard, our study has five implications for the policy reforms. First, more effective ways need to be adopted to improve providers’ knowledge about RUM. Second, action is needed to shift the role of initial diagnosis from village providers to hospital-based providers to allow village providers to assume more responsibility in follow-up visits. Third, although China has implemented the EDL and ZMDP, unnecessary drug prescription is still prevalent, due mainly to inappropriate provider efforts; thus, more research is needed to explore the underlying mechanisms. Fourth, in the short run, the regulation of the prescription of injections appears to be a necessary and effective way to reduce IUM. Finally, given the wide use of CPMs, more research is necessary to assess their appropriateness.

When viewed from a broader perspective, the findings suggest that attention to improving the quality of medical care should focus on providers’ education and training as well as on the governance and management of improving providers’ efforts toward quality care through market-based incentives, regulations, or social accountability approaches.

## Data Availability

The datasets analyzed for this study can be found in the Harvard Dataverse: https://doi.org/10.7910/DVN/U39CHC

## Footnotes

1 1 US$ = 6.7518 yuan (October 2017).

2 Clinical pathways are document-based tools that provide a link between the best available evidence and multidisplinary clinical practice in health care (Rotter et al. (2013). It has been proposed to serve as an intervention to reduce variations in practice and improve patient outcomes in many countries including China (ibid., National Health Commission of the People’s Republic of China, (2016)). In our study, we use the familiarity with the term *clinical pathway* as a proxy that village providers had better clinical knowledge.

3 There are two outliers in this variable. After excluding the outliers, the mean and standard deviation will be 502 and 702, respectively.
