# Supplementary material for: Irrational Use of Medicine in the Treatment of Presumptive Asthma Among Rural Primary Care Providers in Southwestern China
Source: Front Pharmacol. 2022 Feb 15;13:767917. doi: 10.3389/fphar.2022.767917 (PMC8885990; doi:10.3389/fphar.2022.767917)
Supplement: Supplementary file 1 [file DataSheet1.docx]

Supplementary Material

# Appendix SA Provider Survey Form

**Introduction：**Next，We will start filling in the doctor form which focuses on all the doctors responsible for inquiry or prescription (the number of which equals to the total number of “1”answered in the Column e of Part A in the Village Clinic Facility Form).

**A. Basic information about doctor**

**A1. Personal information**

**Introduction:** First, please answer the questions regarding your personal information.

| **Question** | | **Unit / Options** | **Answer** |
| --- | --- | --- | --- |
|  | Gender | 1=Male 2=Female |  |
|  | Ethnic | 1=Han 2=Yi  3=Bai 4=Hani  5=Zhuang 6=Miao  7=Hui 8=Lisu  9= Lahu 10=Wa  11=Naxi 12=Yao  13=Other, please specify_______ |  |
|  | Birth Date | Example：19670120 |  |
|  | Are you native in the village? | 1=Yes 2=No |  |
|  | What’s your responsibility in the clinic? | 1=All the work  2=Medication  3=Public health  4= Other, please specify |  |
|  | When did you first start being a doctor? | Year, such as 1988 |  |
|  | Since then, how many years did you work on other jobs instead of being a doctor（Part-time jobs are not taken into account）? | Years（answer 0 if never worked on other jobs；can be decimal） |  |
|  | Since when have you been working in this clinic（note not be earlier than the birth year） | Year, such as 1997 |  |
|  | What’s your main reason of being a doctor?  （Must read through all the options） | 1=Having a relative or family member being a doctor  2=Learned medicine before  3=There was no doctor in the village  4=Enjoying being a doctor  5=To make a living  6=Elected by the village collective  7=Other, please specify |  |
|  | Do you have any family member or relative being a village doctor before your practice? | 1=Yes 2=No |  |
|  | Do you wish your children or grandchildren to be village doctors in the future? | 1=Yes 2=No |  |
|  | In 2016, of the time for doctor-related work, what percentage was devoted to public health service? (Less than 100) | % |  |
|  | In the last week, how many hours did you spend on doctor-related work? Deduct the time for meals, sleep, leisure and other things. | Hours（Less than 168 hours） |  |

**A2. Education**

| **Question** | | | **Unit / Options** | | | **Answer** | | |
| --- | --- | --- | --- | --- | --- | --- | --- | --- |
|  | What is your highest education level, including adult education?（check the certificate） | | 0=None  1=Elementary school or below（including failed to graduate from junior high school）  2=Junior high school（including failed to graduate from senior high school）  3=Senior high school  4=Vocational high school  5=Junior college  6=College  7=Graduate school or above | | |  | | |
|  | What is your highest achieved qualification certificate? （check the certificate） | | 0=None  1=Village doctor qualification certificate  2=Practicing assistant physician  3=Practicing physician  4=Other, please specify | | |  | | |
|  | Have you received any diploma medical education, including full-time education, in-service education and correspondence education? (check the certificate) | | 1=Yes  2=No **>> Question 9** | | |  | | |
| **Introduction:** Next we want to know about each of your diploma medical education experience, which includes full-time education，in-service education and correspondence education, but excludes training.【Better to check the education certificate to make sure it is diploma education】  **Notice for enumerator**： Question 4 to 8 are dependent on the answer to Question 3. Read each question completely. | | | | | | | |  |
| **Question** | | **Unit / Options** | **Medical Education** | | | | | |
|  |  |  | **The first time** | **The second time** | **The third time** | | **The fourth time** | |
|  | The year completed the education | Year（such as 1990） |  |  |  | |  | |
|  | Is it full-time education, which means studying at school without any work? | 1=Yes 2=No |  |  |  | |  | |
|  | What is the education level?  **（Notice：if the doctor is not sure, enumerator should decide according to the certificate）** | 1=Vocational high school  2=Junior college  3=College  4=Graduate school and above |  |  |  | |  | |
|  | What is the major? | 1=Chinese medicine  2=Western medicine  3=Combination of Chinese and western medicine  4=Public health  5=Nursing  6=No specific major  7=Other, please specify |  |  |  | |  | |
|  | Did you pay for the education fully? | 1=Yes 2=No |  |  |  | |  | |
|  | Have you received any non-diploma medical training before being a doctor? | | 1=Yes 2=No **>> Part A3** | | |  | | |
|  | How many days of non-diploma medical training did you receive before being a doctor? | | Days | | |  | | |

**A3. Training**

**Introduction：**Next we want to know about the face-to-face medical training that you participated in 2016.

|  | Question | 1. Options | 1. Answer | | | | |
| --- | --- | --- | --- | --- | --- | --- | --- |
| 1. 1 | Did you participate in medical trainings that were organized by township hospitals in 2016? | 1=Yes  2=No **>> Question 4** |  | | | | |
| 1. 2 | If yes, how many times did you participate in 2016? | Times |  | | | | |
|  | What were the main contents of the trainings organized by township hospitals in 2016? | 1=Emphasis on medication  2=Emphasis on public health  3=Emphasis on both medication and public health  4=Other, please specify |  | | | | |
| 1. 4 | Did you participate in any face-to-face medical training organized by other institutes other than township hospitals in 2016? (Excluding web-based training) | 1=Yes  2=No **>> Question 23** |  | | | | |
|  | Question | 1. Options | 1. Answer | | | | |
|  |  |  | 1. a. County health department | 1. b. Municipal health department | 1. c. Other institute 1:_______ | 1. d. Other institute 2: _______ | 1. e. Other institute 3:______ |
|  | Have you participated in medical trainings organized by XXX (ask in the order of county health department, municipal health department and other institutes) in 2016? | 1=Yes  2=No **>>Next column** |  |  |  |  |  |
|  | If yes, how many times did you participate in the medical trainings organized by the institute in 2016? | Number of times |  |  |  |  |  |
|  | For the Xth training, what is X? | X | County health department X | Municipal health department X | Other institute 1:_______  X | Other institute 2: _____  X | Other institute 3:______  X |
|  | Which month did the training start on? | Month（such as 7） |  |  |  |  |  |
|  | How long did the training take, excluding commuting time? | Days（allow decimals） |  |  |  |  |  |
| 9.1. | How long did the training actually last, excluding commuting time? (9.1<=9) | Days（allow decimals） |  |  |  |  |  |
|  | Did the training include clinical practice? | 1=Yes 2=No |  |  |  |  |  |
|  | Was the training free? | 1=Yes **>>Question 13**  2=No |  |  |  |  |  |
|  | If not, how much was the fee？ | Yuan |  |  |  |  |  |
|  | Did you **mainly** afford the transportation and accommodation fee caused by the training? | 1=Yes  2=No **>> Question 15** |  |  |  |  |  |
|  | If yes, how much was the transportation and accommodation fee | Yuan |  |  |  |  |  |
|  | Did your clinic operate normally during this training? | 1=Yes **>>Question 17**  2=No |  |  |  |  |  |
|  | How much was it expected to earn during this training if your clinic had operated normally? | Yuan |  |  |  |  |  |
|  | How many times did you consult an expert within the month after the training? | Number of times（answer 0 if none） |  |  |  |  |  |
|  | Do you think the training was helpful for improving medical techniques? | 1=Not helpful at all  2=Basically not helpful  3=A little helpful  4=Helpful  5=Very helpful |  |  |  |  |  |
|  | How much were you willing to pay for the training fee if it was not free? | Yuan |  |  |  |  |  |
|  | How much were you willing to pay for the transportation and accommodation fee for this training? | Yuan |  |  |  |  |  |
|  | Where did the trainers mainly come from? | 1=Doctors from village or community  2=Doctors from township or street office  3=Doctors from county hospitals  4=Doctors from municipal hospitals  5= Doctors from provincial hospitals  6=Other, please specify |  |  |  |  |  |
|  | What were the contents of the training? |  |  |  |  |  |  |
| 22.1 | Hypertension | 1=Yes 2=No |  |  |  |  |  |
| 22.2 | Diabetes | 1=Yes 2=No |  |  |  |  |  |
| 22.3 | Tuberculosis | 1=Yes 2=No |  |  |  |  |  |
| 22.4 | AIDS | 1=Yes 2=No |  |  |  |  |  |
| 22.5 | Mental disorder or mental illnesses | 1=Yes 2=No |  |  |  |  |  |
| 22.6 | Chronic lung diseases | 1=Yes 2=No |  |  |  |  |  |
| 22.7 | Coronary heart diseases | 1=Yes 2=No |  |  |  |  |  |
| 22.8 | Pediatric diarrhea | 1=Yes 2=No |  |  |  |  |  |
| 22.9 | Proper use of antibiotics | 1=Yes 2=No |  |  |  |  |  |
| 22.10 | Chinese herbal medicine | 1=Yes 2=No |  |  |  |  |  |
| 22.11 | TCM physiotherapy | 1=Yes 2=No |  |  |  |  |  |
| 22.12 | Physical examination | 1=Yes 2=No |  |  |  |  |  |
| 22.13 | Emergency and first aid | 1=Yes 2=No |  |  |  |  |  |
| 22.14 | Gynecological diseases | 1=Yes 2=No |  |  |  |  |  |
| 22.15 | Andrology | 1=Yes 2=No |  |  |  |  |  |
| 22.16 | Orthopedic diseases（such as fractures and osteoarthritis） | 1=Yes 2=No |  |  |  |  |  |
| 22.17 | Skin diseases | 1=Yes 2=No |  |  |  |  |  |
| 22.18 | Surgical abdomen | 1=Yes 2=No |  |  |  |  |  |
| 22.19 | Nursing | 1=Yes 2=No |  |  |  |  |  |
| 22.20 | Rhinitis | 1=Yes 2=No |  |  |  |  |  |
| 22.21 | Child epilepsy | 1=Yes 2=No |  |  |  |  |  |
| 22.22 | Thyroid diseases | 1=Yes 2=No |  |  |  |  |  |
| 22.23 | Other, please specify__________ | 1=Yes 2=No |  |  |  |  |  |
| 22.24 | Other, please specify__________ | 1=Yes 2=No |  |  |  |  |  |
| 22.25 | Other, please specify__________ | 1=Yes 2=No |  |  |  |  |  |
| 22.26 | Other, please specify__________ | 1=Yes 2=No |  |  |  |  |  |
| 22.27 | Other, please specify__________ | 1=Yes 2=No |  |  |  |  |  |
| 22.28 | Other, please specify__________ | 1=Yes 2=No |  |  |  |  |  |

| **Question** | | **Unit / Options** | | **Answer** |
| --- | --- | --- | --- | --- |
|  | If possible, what kind of training do you want most? （Excluding web-based training）？  **（Single choice）**  **（Let the doctor know all the options）** | 0=None **>> Question 28**  1= Hypertension 13= Emergency and first aid  2= Diabetes 14= Gynecological diseases  3= Tuberculosis 15= Andrology  4= AIDS 16= Orthopedic diseases（such as  fractures and osteoarthritis）  5= Mental disorder or mental illnesses 17= Skin diseases  6= Chronic lung diseases 18= Surgical abdomen  7= Coronary heart diseases 19= Nursing  8= Pediatric diarrhea 20= Rhinitis  9= Proper use of antibiotics 21= Child epilepsy  10= Chinese herbal medicine 22= Thyroid diseases  11= TCM physiotherapy 23= Other, please specify  12= Physical examination | |  |
|  | For your most wanted training, in what way of delivery do you most prefer?  **（Single choice）（Let the doctor know all the options）** | | 1=Seminar lecture  2=Interactive teaching  3=Scenario simulation  4=Clinic practice  5=Other, please specify |  |
|  | For your most wanted training, how many days at most are you willing to spend on it off-job? | | Days（allow decimals） |  |
| 1. 、 | For your most wanted training, how much at most are you willing to pay? Only for the training fee, not including the transportation and accommodation fee. | | Yuan |  |
|  | For your most wanted training, are you willing to pay for the transportation and accommodation fee? | | 1=Yes 2=No |  |
|  | Did you participate in any training at the county level or above from 2013 to 2015? | | 1=Yes 2=No |  |
|  | Did you take any internship at a upper-level hospital from 2013 to 2016?（Excluding trainings mentioned above）？ | | 1=Yes 2=No |  |

**Distance training**

**Introduction：** Next we want to know about the distance trainings that you participated in 2016.

| **Question** | | **Unit / Options** | | **Answer** |
| --- | --- | --- | --- | --- |
|  | Did you participate in any form of distance online training that is related to your clinical work in 2016? | | 1=Yes 2=No **>>Question 40** |  |
|  | If yes, how many times did you participate in? | | Number of times |  |

|  | |  |  | The first time | The second time | The third time | The fourth time |
| --- | --- | --- | --- | --- | --- | --- | --- |
|  | How did you obtain the opportunity to participate in this training? | | 1=Upper-level authorities 2=Other doctors  3=The Internet 4=Other, please specify |  |  |  |  |
|  | Where did you participate in this training? | | 1=Home / clinic 2=Village committee office  3=Township hospital 4=Other, please specify |  |  |  |  |
|  | What are the contents of the training？ | |  |  |  |  |  |
| 34.1 | Hypertension | | 1=Yes 2=No |  |  |  |  |
| 34.2 | Diabetes | | 1=Yes 2=No |  |  |  |  |
| 34.3 | Tuberculosis | | 1=Yes 2=No |  |  |  |  |
| 34.4 | AIDS | | 1=Yes 2=No |  |  |  |  |
| 34.5 | Mental disorder or mental illnesses | | 1=Yes 2=No |  |  |  |  |
| 34.6 | Chronic lung diseases | | 1=Yes 2=No |  |  |  |  |
| 34.7 | Coronary heart diseases | | 1=Yes 2=No |  |  |  |  |
| 34.8 | Pediatric diarrhea | | 1=Yes 2=No |  |  |  |  |
| 34.9 | Proper use of antibiotics | | 1=Yes 2=No |  |  |  |  |
| 34.10 | Chinese herbal medicine | | 1=Yes 2=No |  |  |  |  |
| 34.11 | TCM physiotherapy | | 1=Yes 2=No |  |  |  |  |
| 34.12 | Physical examination | | 1=Yes 2=No |  |  |  |  |
| 34.13 | Emergency and first aid | | 1=Yes 2=No |  |  |  |  |
| 34.14 | Gynecological diseases | | 1=Yes 2=No |  |  |  |  |
| 34.15 | Andrology | | 1=Yes 2=No |  |  |  |  |
| 34.16 | Orthopedic diseases（such as fractures and osteoarthritis） | | 1=Yes 2=No |  |  |  |  |
| 34.17 | Skin diseases | | 1=Yes 2=No |  |  |  |  |
| 34.18 | Surgical abdomen | | 1=Yes 2=No |  |  |  |  |
| 34.19 | Nursing | | 1=Yes 2=No |  |  |  |  |
| 34.20 | Rhinitis | | 1=Yes 2=No |  |  |  |  |
| 34.21 | Child epilepsy | | 1=Yes 2=No |  |  |  |  |
| 34.22 | Thyroid diseases | | 1=Yes 2=No |  |  |  |  |
| 34.23 | Other, please specify__________ | | 1=Yes 2=No |  |  |  |  |
| 34.24 | Other, please specify__________ | | 1=Yes 2=No |  |  |  |  |
| 34.25 | Other, please specify__________ | | 1=Yes 2=No |  |  |  |  |
| 34.26 | Other, please specify__________ | | 1=Yes 2=No |  |  |  |  |
| 34.27 | Other, please specify__________ | | 1=Yes 2=No |  |  |  |  |
| 34.28 | Other, please specify__________ | | 1=Yes 2=No |  |  |  |  |
|  | |  |  | The first time | The second time | The third time | The fourth time |
|  | How many training hours did the training have? | | Number of training hours |  |  |  |  |
|  | How many hours did one training hour have? | | Minutes |  |  |  |  |
|  | Training fee（answer 0 if none），excluding transportation and accommodation fee | | Yuan |  |  |  |  |
|  | Did you mainly afford the transportation and accommodation fee for this training? | | 1=Yes 2=No  3=No transportation and accommodation fee |  |  |  |  |
|  | Do you think the training was helpful for improving medical techniques? | | 1=Not helpful at all  2=Basically not helpful  3=A little helpful  4=Helpful  5=Very helpful |  |  |  |  |

|  | **Question** | **Unit / Options** | **Answer** |
| --- | --- | --- | --- |
|  | If possible, what kind of online training do you want most?  **（Single choice）**  **（Let the doctor know all the options）** | 0=None **>> Question 42**  1= Hypertension 13= Emergency and first aid  2= Diabetes 14= Gynecological diseases  3= Tuberculosis 15= Andrology  4= AIDS 16= Orthopedic diseases（such as fractures and osteoarthritis）  5= Mental disorder or mental illnesses 17= Skin diseases  6= Chronic lung diseases 18= Surgical abdomen  7= Coronary heart diseases 19= Nursing  8= Pediatric diarrhea 20= Rhinitis  9= Proper use of antibiotics 21= Child epilepsy  10= Chinese herbal medicine 22= Thyroid diseases  11= TCM physiotherapy 23= Other, please specify  12= Physical examination |  |
|  | For your most wanted online training, how much at most are you willing to pay? | Yuan |  |
|  | Which mode of training do you want most? | 1=Face-to-face training  2= Web-based training  3=Both  4=Neither  5=Other, please specify |  |

**B. Admissions**

**Introduction：** The following are some situations during an admission.

| **Question** | | **Unit / Options** | **Answer** |
| --- | --- | --- | --- |
|  | What is the percentage of your patients who need injection or intravenous infusion? | %（answer 0 if none） |  |
|  | What is the percentage of responsibility for doctor to make sure patients to take the medicine according to medical advice?（Do not explain） | %（answer 0 if none） |  |
|  | Basic on your estimation, what is the percentage of your patients who do not take the medicine according to medical advice? | %（answer 0 if none） |  |
|  | Basic on your estimation, what is the percentage of your patients who ask you to prescribe antibiotics directly? | %（answer 0 if none） |  |
|  | Basic on your estimation, what is the percentage of your patients who want you to prescribe antibiotics but did not ask so directly? | %（answer 0 if none） |  |
|  | How would you prescribe if a patient is poor?*（Notice：do not read the options）* | 1=Prescribing based on economic condition  2=Prescribing based on medical condition  3=Taking both conditions into consideration |  |
|  | When a patient walk in, what is the percentage of certainty that you can conclude his/her disease without any inquiry or inspection? （Do not explain） | %（answer 0 if none） |  |

**C. Treatment for common symptoms**

**Introduction：** Next we want to know about if you have had patients with the following symptoms during the last months?

*Notice for enumerator: if the doctor says he/she don’t prescribe antibiotics when answering Question b, enumerator should emphasize that “We are not asking the real situation but your estimation on the possibility of curing a patient by taking antibiotics”. The doctor need to answer Question b even if his/her answer to Question a is 0.*

| **Symptom** | | 1. **How many patients had such symptom during the last month?（Number of visits，answer 0 if none）** | **b. Based on your estimation, what is the percentage of possibility to cure a patient with such symptom by taking antibiotics?（%）** |
| --- | --- | --- | --- |
|  | Diarrhea |  |  |
|  | Difficult to breathe |  |  |
|  | Having a headache and a hot face |  |  |
|  | Running at the nose, coughing and feeling of malaise |  |  |

**D. Assessment**

**introduction：Next we want to know about the assessments that were conducted by the upper-level authorities in 2016, including time, contents, rewards and punishments.**

| **Question** | | **Unit / Options** | **Answer** |
| --- | --- | --- | --- |
| 1. 1. | Did upper-level authorities assess you in 2016? | 1=Yes 2=**No >>Part E** |  |
| 1. 2. | How often is the assessment? | 1=Once a year 2=Once a half year  3=Once a season 4=Once a month  5=Once a week  6=Other, please specify |  |
| 1. 3. | Did upper-level authorities assess you on public health service in 2016? | 1=Yes 2=No |  |
| 1. 4. | Did upper-level authorities assess you on prescription in 2016? | 1=Yes 2=No |  |
| 1. 5. | Did upper-evel authorities assess you on proper use of antibiotics in 2016? | 1=Yes 2=No |  |
| 1. 6. | Will you receive monetary rewards if the assessment result is good? | 1=Yes 2=No |  |
| 1. 7. | Will your money be deducted if you fail to pass the assessments? | 1=Yes 2=No |  |

**E. Income**

**Introduction：** Next we want to know about your income

| **Question** | | **Unit / Options** | **Answer** |
| --- | --- | --- | --- |
|  | What was your basic salary in 2016? Basic salary means the fixed salary paid by upper-level authorities, which is irrelevant to the workload and assessments. | Yuan/Year（answer 0 if none） |  |
|  | How much of the net income of the village clinic was distributed to you in 2016?  （If the village clinic has more than one staff and all of them are interviewed, the answers from them should add up to 100%.） | % |  |
|  | Do you have any other job apart from being a doctor? | 1=Yes 2=No>> **Part F** |  |
|  | What other jobs do you have?  **（Read all the options; multiple choices, use comma to separate the answers）** | 1=Farming but all the agricultural products are for family consumption（no need to ask income from this）  2=Farming and selling agricultural products  3=Doing part-time jobs  4=Doing business  5=Village cadre  6=Other, please specify |  |
|  | What was your income from other jobs apart from that from the clinic in 2016?（For example: farming, doing part-time jobs or other jobs mentioned above.） | Yuan |  |

**F. Phone and the Internet**

**Introduction：**Next we want to know about your source of information.

| **Question** | | **Unit / Options** | **Answer** |
| --- | --- | --- | --- |
|  | Do you have a smart phone (Android/iPhone) or a computer (including computer at workplace or at home)？ | 1=Both 2=Only the smart phone  3=Only the computer  4=Neither>> **Question 11** |  |
|  | If yes, is it connected to the Internet? | 1=Yes 2=No**>> Question 11** |  |
|  | Do you know how to use computer? | 1=Yes 2=No |  |
|  | Do you use QQ on cell phone or computer? | 1=Yes 2=No |  |
|  | Do you use WeChat on cell phone or computer? | 1=Yes 2=No |  |
|  | How often do you search for medical information on your phone or computer? | 1=One or more times a week  2=One to three times a month  3=One to five times every half year  4=No more than once a year |  |
|  | Do the village doctors in your county or town have a WeChat or QQ group? | 1=Neither>> Question 9  2=Only among the doctors in the town  3=Only among the doctors in the county4=Both |  |
|  | Do you discuss medical knowledge with other village doctors in the group mentioned above? | 1=No  2=Yes, but only asking questions  3= Yes, but only answering questions  4=Yes, both asking and answering questions |  |
|  | Do the village doctors have a WeChat or QQ group with doctors from township hospitals or county hospitals? | 1=Neither>> Question 11  2=Only with the doctors from township hospitals  3=Only with the doctors from county hospitals  4=Both |  |
|  | Do you discuss medical knowledge with doctors from township hospitals or county hospitals in the group mentioned above? | 1=No  2=Yes, but only asking questions  3= Yes, but only answering questions  4=Yes, both asking and answering questions |  |
|  | If encountering a symptom that you don’t know how to treat, what is your major way to get an answer?（Read all the options） | 1=Enquiring in WeChat or QQ group  2=Calling an acquainted doctor  3=Searching on the Internet by using phone or computer  4=Looking up in medicine books  5=Other, please specify  6=Doing nothing（Including transferring） |  |

**G．Antibiotics**

**Introduction：** Next we want to know about your views on antibiotics

**Notice for enumerator：** Let the doctor choose the answers in Part G2. Do not read or explain anything. Help the doctor use the iPad.

**G1. Use of antibiotics**

| **Question** | | **Unit / Options** | **Answer** |
| --- | --- | --- | --- |
|  | In all the antibiotics that you prescribed in 2016, what are the percentages of their reasons：（**Notice：1+2+3+4=100%**） | | |
|  | 1.1 For treating the disease directly | % |  |
|  | 1.2 For preventing the current disease from exacerbating | % |  |
|  | 1.3 For getting the diagnosis results of the current disease | % |  |
|  | 1.4 Other reasons | % |  |
|  | What is your major source of information on the use of antibiotics? | 1= Based on my clinical experience  2= Materials / policies given by upper-level authorities  3=Trainings by health department  4=Materials from pharmaceutical firms  5=Salesperson from pharmaceutical firms  6=Other village doctors  7=Textbooks  8=The Internet |  |

**G2. Opinions on antibiotics**

**Introduction：** Next you will answer the questions by yourself. We will not explain the questions or options. **They are all single choice questions. Please read and answer carefully.**

|  | One who has a cold will recover quickly if taking antibiotics. | 1=True 2=Wrong |  |
| --- | --- | --- | --- |
|  | Under what condition should a patient stop taking antibiotics? | 1=When feeling better  2=When having taken all prescribed antibiotics  3=Do not know |  |
|  | For those uncertain cases, it’s best to use antibiotics as part of the treatment. | 1=Completely agree  2= Somehow agree  3= Neutral  4= Somehow disagree  5= Completely disagree |  |
|  | In order to let patients trust on my medical skills, I will prescribe antibiotics even it is unnecessary. | 1=Completely agree  2= Somehow agree  3= Neutral  4= Somehow disagree  5= Completely disagree |  |
|  | Patients often ask doctors to prescribe antibiotics. | 1=Completely agree  2= Somehow agree  3= Neutral  4= Somehow disagree  5= Completely disagree |  |
|  | It’s hard not to prescribe antibiotics when patients are asking so ,even though I don’t think they need them. | 1=Completely agree  2= Somehow agree  3= Neutral  4= Somehow disagree  5= Completely disagree |  |

# Appendix SB The Clinic Survey Form

**Dear doctor：hello！**

Thank you very much for participating in the survey of “Yunnan Health Service Research”. The project is funded by “Health and Hope Fund” of the Business Development Center of the Red Cross Society of China (RCSC) (Beijing) and UCB (Belgium). We are research team from Peking University. The project aims to provide policy recommendations for improving rural healthcare service by understanding the status of primary healthcare in the rural area. Your support and cooperation are very important to us. We guarantee that your answers will only be used for research purposes and kept strictly confidential. Please do not worry. Thank you for your cooperation.

**Notice for enumerator：**

1）Read questions and options completely. Please write the doctor’s answers in the answer column. Unless otherwise specified, questions refer to the situation in 2017 (this year).

2）If a question requires number, please write the specific number instead of a range. If a doctor is used to answering a range, tell him /her “We need a specific number instead of a range. Please try to answer a specific number”. If the doctor still cannot give a specific number after 3 reminds, you should take the average of the range.

3）If the doctor does not know how to answer, write 888 and his / her reason for not knowing how to answer. If there is a special case and you don't know how to write, write 999 and the special case.

4）You do not need to read the texts in the bracket. Read them to the doctor if he or she has a question.

**A. List of clinic staff**

**Introduction:** Next we want to know about the staff in the clinic.

| **a．Name of the staff** | | **b. Gender**  **1=Male**  **2=Female** | **c. Age（years old）** | **d．Responsibility**  **1=Consultation and prescription**  **2=Consultation and prescription but only on Chinese herbal medicine >> Next staff**  **3=Not for consultation and prescription >>Next staff** | **e. Did he / she answer the doctor form?（If the staff has not been working in the clinic for more than 4 months, then he / she doesn't need to answer the doctor form）**  **1=Yes🡺Next staff**  **2=No** | **Notes**  **(Reasons that he / she didn’t answer the doctor form）** |
| --- | --- | --- | --- | --- | --- | --- |
| 1. 听 |  |  |  |  |  |  |
| 1. 体 |  |  |  |  |  |  |

**B. Basic information about the clinic**

**Introduction:** Next we want to know about the basic information about the clinic.

| **Question** | | **Unit / Options** | **Answer** |
| --- | --- | --- | --- |
|  | How many of the doctors responsible for consultation and prescription are township hospital formal establishment staff?（The answer should be less than or equal to the sum of “1”s answered for Question d in Part A） | Number of staff  **>>If 0，then go to Question 3** |  |
|  | Names of the township hospital formal establishment staff |  | |
|  | Is your clinic a designated medical institution of NCMS? | 1=Yes  2=No>> Part C |  |
|  | Can the villagers who are participants of NCMS get reimbursed from NCMS for the cost of visits to your clinic? | 1=Yes 2=No |  |

**C. Service**

**C1. Basic information**

**Introduction:** Next we want to know about the basic information on the clinic service.

| **Question** | | **Unit / Options** | **Answer** |
| --- | --- | --- | --- |
|  | Including your clinic, how many clinics are there within 5 km of your clinic as long as one can see a doctor there? | Number of clinics |  |
|  | How many pharmacies are there within 5 km of your clinic, including drugstores and excluding clinics? （Answer 0 if none） | Number of pharmacies |  |
|  | How many permanent residents are there within 5 km of your clinic, including local residents and migrants? | Number of person |  |
|  | How many of the permanent residents population within 5 km of your clinic visit your clinic? | % |  |
|  | How many patients visited your clinic in the last month? | Number of visits |  |
|  | Among them, how many did you recommend to be referred to a superior hospital?（6<5） | Number of visits |  |
|  | How many patients whom you didn’t know visited your clinic in the last month? | Number of person |  |
|  | Does your clinic charge a general consultation fee, including that being exempted or deducted for participants of NCMS? | 1=Yes, we charge for all patients  2= Yes, we charge for some patients （Please elaborate）  3=No **>>Question 10** |  |
|  | If yes, how much is the general consultation fee? (Check if the answer is the amount after exemption deduction) | Yuan/visit |  |
|  | Does your clinic use intramuscular injection? | 1=Yes 2=No **>> Question 12** |  |
|  | How many patients received intramuscular injection in the last month? (11<5) | Number of visits |  |
|  | Does your clinic use intravenous injection? | 1=Yes 2=No **>>Question 14** |  |
|  | How many patients received intravenous injection in the last month?？(13<5) | Number of visits |  |
|  | Did your clinic provide public health service in 2016? | 1=Yes 2=No **>> Part C2** |  |
|  | How many administrative villages were your clinic responsible for the public health service in 2016? | Number of villages  （allow decimals，such as 0.5） |  |
|  | What is the population of the administrative villages mentioned above? | Number of person |  |

**C2. Medical equipment and use**

**Introduction:** Next we want to know about the medical equipment and their use. Please show me the equipment when I asked about a specific equipment.

**Notice for enumerator:** Note all equipment is to be seen as true. Enumerator should confirm with the doctor that the equipment is available for use. An equipment cannot be counted if it breaks down and the doctor has no plan to fix it.

| **Name of the equipment** | **a．Do you have this equipment?**  **1=Yes**  **2=No>>Next line** | **b．Is it distributed by upper-level authorities for free?**  **1=Yes**  **2=No** | **c．Was it used in the last week?**  **1=Yes**  **2=No** |
| --- | --- | --- | --- |
| 1. Stethoscope |  |  |  |
| 1. Thermometer |  |  |  |
| 1. Sphygmomanometer |  |  |  |
| 1. Tongue spatula |  |  |  |
| 1. Tourniquet |  |  |  |
| 1. Treatment disc |  |  |  |
| 1. Visiting box |  |  |  |
| 1. High temperature sterilizer with pressure gauge |  |  |  |
| 1. UV disinfection lamp |  |  |  |
| 1. Debridement suture package |  |  |  |
| 1. Hospital bed |  |  |  |
| 1. Sputum suction |  |  |  |
| 1. Refrigerated bag |  |  |  |
| 1. Oxygen bag or bottle |  |  |  |
| 1. Blood routine tester |  |  |  |
| 1. Blood glucose monitor |  |  |  |
| 1. Height and weight meter |  |  |  |
| 1. Trash can with lid |  |  |  |
| 1. Computer |  |  |  |
| 1. Printer |  |  |  |
| 1. Medicine cabinet |  |  |  |
| 1. Vacuum cupping |  |  |  |
| 1. Acupoint therapy instrument |  |  |  |
| 1. Moxibustion box |  |  |  |
| 1. Massage bed |  |  |  |
| 1. Multi-parameter health tester |  |  |  |
| 1. Freezer or refrigerator |  |  |  |
| 1. Specific electromagnetic wave treatment instrument or magic lamp |  |  |  |
| 1. Other 1, please specify_____ |  |  |  |

**D. Visit records**

**Introduction:** Next we want to know about the visit records kept in your clinic. Please take out all the documents related to patient visits so that we can look at it together.

**Notice for enumerator:** For Questions 1 to 11, ask the doctor what records and documents are related to patient visits and ask him / her to take them out. Answer the questions for all kinds of records. Records specific to a few special patients are not counted (for example, only keeping the record for patients who owe money).

| **Question** | | **Unit / Options** | **Answer** |
| --- | --- | --- | --- |
|  | Do you keep records for patient visits, such as patient information, outpatient logs and prescriptions?（If only keeping records on name of patient and visit date, then it is not counted and choose 2=No for this question） | 1=Yes 2=No **>>Question 8** |  |
|  | In what form is the record kept? | 1=Paper records  2=Electronic records 3=Both |  |
|  | Dose the record include personal information of patients? (Name, gender, age, etc.) | 1=Yes 2=No |  |
|  | Does the record include a patient’s main symptoms? | 1=Yes 2=No |  |
|  | Does the record include a patient’s past medical history? | 1=Yes 2=No |  |
|  | Does the record include the diagnosis of a patient? | 1=Yes 2=No |  |
|  | Does the record include the treatment plan (prescription) for a patient? | 1=Yes 2=No |  |
|  | Did upper-level authorities inspect the medical records or prescription kept in the clinic in 2016? | 1=Yes 2=No **>>Part E** |  |
|  | How often were you inspected in 2016? | 1=In three months  2=In three to six months  3=In six to nine months  4=In nine months to a year  5=More than a year |  |
|  | Would there be a monetary penalty if an obvious error was found? | 1=Yes 2=No |  |
|  | Would there be an administrative penalty if an obvious error was found, such as verbal criticism and notice of criticism? | 1=Yes 2=No>>Part **E** |  |
|  | What administrative penalties will be imposed if an obvious error was found? (Multiple choices, read all the options) | 1=Verbal criticism  2=Notice of criticism  3=Revocation of Village Doctor Practice Certificate  4=Revocation of Village Clinic Business License  5=Other, please specify |  |

**E. Medicine and use**

**Introduction:** Next we want to know about the medicine that your clinic has and their use.

| **Question** | | **Unit / Options** | **Answer** |
| --- | --- | --- | --- |
|  | By the end of 2016, how many kinds of western medicines did your clinic have? | Number of kinds |  |
|  | By the end of 2016, how many kinds of Chinese patent medicines did your clinic have? | Number of kinds |  |
|  | By the end of 2016, how many kinds of Chinese herbal medicines did your clinic have? | Number of kinds |  |
|  | By the end of 2016, what was the total value of the medicines that your clinic had? | Yuan |  |
|  | By the end of 2016, how much of the medicines was purchased by your clinic based on the kinds of medicines? Excluding the medicines purchased by National Basic Medicine Platform and township hospitals. | %（Answer 0 if none） |  |
|  | By the end of 2016, how much of the medicines was purchased by your clinic based on purchasing value? Excluding the medicines purchased by National Basic Medicine Platform and township hospitals. | %（Answer 0 if the answer for Question 5 is 0） |  |
|  | Did your clinic implement the zero-difference rate policy? (That’s the sale price of medicine equals to its purchase price) | 1=Yes  2=No**>> Part F** |  |
|  | Did upper-level authorities evaluate the implementation of the zero-difference rate policy of your clinic in 2016? | 1=Yes  2=No |  |

**F. Location of the clinic**

**Introduction:** Last, we want to know about the geographic location and economy of the village.

| **Question** | | **Unit / Options** | **Answer** |
| --- | --- | --- | --- |
|  | How far is the clinic from the township hospital? | Km |  |
|  | Is there a shuttle bus or van from the village to the township hospital operating every day? | 1=Yes  2=No>>Question 4 |  |
|  | How much does a one way trip cost if a patient takes a shuttle bus or van from the village to the township hospital (renting a private car is not counted)?（The largest amount） | Yuan |  |
|  | How far is the clinic from the county hospital that villagers visit the most frequently? | Km |  |
|  | Is there a shuttle bus or van from the village to the county hospital that villagers visit the most frequently operating every day? | 1=Yes  2=No>>Question 7 |  |
|  | How much does a one way trip cost if a patient takes a shuttle bus or van from the village to that county hospital (renting a private car is not counted)?（The largest amount） | Yuan |  |
|  | What is the average daily wage for a 50-year-old male who works on odd jobs (not a skilled worker)? | Yuan |  |
|  | What is the average daily wage for a 50-year-old female who works on odd jobs (not a skilled worker)? | Yuan |  |

**G. Common diseases management**

**Introduction:** Next, we want to know your how your clinic manage common diseases.

**Notice for enumerator:** Ask the questions by column. Insert the diseases into the questions when asking.

|  | **tuberculosis** | **Type 2 diabetes** | **hypertension** | **Pharyngitis** | **Pediatric diarrhea** | **Asthma** | **Chronic lung disease** | **Angina pectoris** |
| --- | --- | --- | --- | --- | --- | --- | --- | --- |
| 1. Was your clinic responsible for then management of patients with XX in 2016?（1=Yes 2=No**>>Question 3**） |  |  |  |  |  |  |  |  |
| 2. How many patients with XX you're your clinic manage in 2016?（Answer 0 if none） |  |  |  |  |  |  |  |  |
| 3. How many suspected cases of XX did your clinic find in 2016?【Go to next disease if none】 |  |  |  |  |  |  |  |  |
| 4. Among them, how many did you recommend referral (including verbal referral) no matter patient did go or not? |  |  |  |  |  |  |  |  |
| 5. Among them, how many did you reported to upper-level authorities? |  |  |  |  |  |  |  |  |

**H. Income of 2016**

**Introduction:** Next we want to know about the income of 2016. Let’s calculate.

**Notice for enumerator:** If the doctor’s answers to Question 5 and 6 in Village Clinic Facility Form 1 are not zero, which means the medicines that the doctor purchased are not zero, then there must exists net income from non-zero rate medicines. (If there is a hired doctor, answer to Question 7 should include the wages for him/her. The wage is included in the net income of the clinic and the clinic distributes its total income.)

| **Questions** | | **Unit / Options** | **Answer** |
| --- | --- | --- | --- |
|  | Subsidy for public health service | Yuan |  |
|  | Subsidy for implementation of the zero-mark-up policy | Yuan |  |
|  | Total of the fixed salaries that all the staff received from county health department | Yuan |  |
|  | Net income from medication, excluding medicines. | Yuan |  |
|  | Net income from non-zero-mark-up medicines | Yuan |  |
|  | Other income（the income is not counted if it is from doctor’s farming or other things irrelevant to the clinic, please specify__**__** | Yuan |  |
|  | Total income of the clinic  （First ask the total income of the clinic. Then compare the amount with the summation of the answers for Question 1 to 6. If the amounts are inconsistent, ask for the reasons. 7=1+2+3+4+5+6） | Yuan |  |

# Appendix SC1 Script of Unannounced Standardized Patient Visit

**Background**

Female patient: Jing Liu, 20 years old.

1. Jing Liu, 20 years old, born in 1997. She is from a neighboring village. She is not married. She does odd jobs nearby and sometimes helps at home.
2. She felt a sense of out of breath as early as a year ago.
3. In the past two or three months, she felt that she gasped and was not able to breathe enough every day and about once a week. It lasted 20-30 minutes each time.
4. When She finished dinner and cleaned the house last night, she suddenly felt out of breath. She gasped for an hour but gradually felt better after an hour. She felt very tired and exhausted, and then she just fell asleep. Compared with the previous situation, this time she felt more severe and the gasp lasted longer, so she planned to see the doctor.
5. When she is sick, her throat feels like whistling when breathe in and out, and she is more likely to gasp during the night. She also coughs, dry cough when she felt out of breath, and cough more at night. Generally, it is easy to occur when there are a lot of dust while cleaning the house or the road. During the gasping, she found that drinking a cup of hot water would get better. She has an older brother at home, 27 years old. Jing Liu's elder brother had similar problems with breathing difficulties in the past few years, and he had already started taking medication, but he didn't know what wrong or what medicine he was taking. Others are healthy. Jing Liu's mother said that she often coughed when she was a child, but Jing Liu did not remember such a situation.
6. She never drinks or smokes. She mainly cooks at home and eat the same as everyone else.
7. She has no other physical problems.

| **Opening remark: Doctor, I feel that I do not have enough breath, and I am out of breath. The symptom is not improved after taking medicine, so I come and have a look.** | | |
| --- | --- | --- |
|  | **Questions the doctor may ask** | **Patient’s answers** |
|  | When did you gasp recently? Are you gasping now?  How many days has it been? | I had a gasp last night |
|  | When did you gasp last night? What time did you gasp? | After dinner last night |
|  | What did you do when you were gasping last night? What did you do yesterday? | When I was cleaning the house last night, I suddenly felt out of breath. |
|  | How long did you gasp last night? | An hour last night |
|  | Was it a bad gasp last night? | An hour last night |
|  | What was the difference between the gasp last night and before? | It took longer and more severe this time |
|  | How did you recover last night? | I gradually felt better after an hour |
|  | Did you clean normally or clean the old stuffs (lots of dusts) last night? Did you gasp after cleaning or within cleaning? | Just when I was cleaning the house |
|  | When did it start? | As early as one year ago |
|  | How long has it been so breathless? How many days has it been? Was there such a condition before? | As early as one year ago |
|  | How often does the breathless condition occur? When did the last one happens before yesterday? Are you sick intermittently? | In the past two or three months, about once a week. But I felt out of breath every day last week. |
|  | Has it changed in recent compared to what it was before? Has it been serious recently? | It has been more frequent in the last 2 or 3 months, and it has been worse last week. |
|  | How long did you gasp before? | 20 to 30 minutes each time before this time |
|  | Was your gasp serious before? | 20 to 30 minutes each time before this time |
|  | Was it difficult to breathe when gasping? Did you breathe quickly when you were gasping | I felt out of breath when gasping |
|  | Was there a special sound when breathing? Was there a sound in your throat? | I felt like whistling in my throat when I breathe |
|  | When gasping, did you feel hard to breathe out or breathe in? | Both |
|  | Did you cough? | Yes |
|  | How long is the cough? | I cough when I can't breathe, but don't cough when I am not breathing |
|  | Did you cough or gasp first? | I cough when I can't breathe, but don't cough when I am not breathing |
|  | Is the cough bad? | I cough when I can't breathe, but don't cough when I am not breathing |
|  | Did you sputum? | Just dry cough and not sputum |
|  | Did you cough up blood? | No |
|  | Did you have sore throat? | No |
|  | Was your throat dry? | No |
|  | Is this situation worse in the morning or at night? When is the day more serious? | It is more likely to be short of breath at night |
| 1. 、 | Is it easier to happen in winter? | No, just as normal |
|  | Did you gasp during the day? | Sometimes I gasp, and I felt out of breath more often at night. |
|  | Did you gasp before or after sleeping? | Gasping before going to bed, and it is more likely to be short of breath at night |
|  | Is there any symptom of upper respiratory tract infection (cold, sneezing, blocked nose)? | No |
|  | Under what circumstances did it happen before? What were you doing every time it happens? | When cleaning the house or when there is much dust on the road |
|  | Can you stay in a dusty environment? | I can’t stay in such an environment because I felt easier to gasp |
|  | Are you allergic to anything? | No |
|  | Is there any smell that cause allergies? | No |
|  | Is there any pollen allergy? | No |
|  | Will there be onsets/allergies from contact with animals? | No |
|  | How does the symptom disappear/relieve/improve? | I get better with a glass of hot water |
|  | How did it get better last night? | I feel better in an hour  If the doctor asks, ‘did you drink hot water last night?’  Answer: I drank it, but it didn't work |
|  | Have you seen a doctor before? Have you infused before? | No |
|  | Why didn't you see a doctor before, but do this time? Is it more serious this time? | I felt worse last night, and I felt out of breath for an hour. |
|  | Do you feel tired/exhaust/lack of energy? | Tire and exhaust |
|  | Any lung illness? | I don’t know, it is just out of breath |
|  | Do you have fever? | No |
|  | Do you have chest pain? | No |
|  | Do you feel flustered? | No |
|  | Has the weight changed? Are you thinner? Did you lose weight? | No |
|  | Do you sweat at night (sweat a lot when you fall asleep)? | No |
|  | Do you have stomachache? | No |
|  | What do you usually eat? | I mainly eat at home like everyone else does |
|  | Do you smoke? | No |
|  | Do you drink? | No |
|  | Did you have this symptom when you were a child? | My mother said that I used to cough when I was a child young, but I can’t remember it. |
|  | Does anyone in the family have the same symptom? | My older brother has it as well |
|  | Has your older brother visited the hospital? | Probably |
|  | What is your older brother's disease? | Not sure about the specific illness |
|  | Does your older brother take medicine? | Yes |
|  | What medicine did your older brother take? | Not sure about the specific illness and don’t know what specific medicine he takes |
|  | Apart from your older brother, is there anyone else in the family who has the same symptom? | No |
|  | Name | Jing Liu |
|  | Do you have health insurance? | I didn’t bring my health insurance record |
|  | What is your family’s financial situation? | Normal |
|  | Are you caught in the rain？ | No |
|  | When is your date of menstruation？ | normal menstruation, the 15th |
|  | What is your job? | Doing odd jobs nearby |
|  | Have you taken any medicine? | Yes, some anti-inflammatory drugs |
|  | What medicine did you take? /What anti-inflammatory drug dud you take? | The medicine was bought by family members, but I don’t remember the details |
|  | Why taking anti-inflammatory drugs? | We have this at home, so I want to see if I can recover after taking it |
|  | When did you take the medicine? | I started taking it 5 days ago |
|  | Did you get better after taking it? | I didn’t feel improved. |
|  | How did you take it? How many did you take? | I took 2 times a day, 2 pills each time |
|  | Where did you buy the medicine? When did you buy the medicine? | The medicine was bought by family members, but I don’t remember the details |
|  | Did you take any other medicines? | No |
| **Examinations or tests** | | |
|  | Non-intrusive examinations or tests | OK (The patient goes for a non-intrusive examination or test) |
|  | Intrusive examinations or tests (X-ray, color Doppler ultrasound, CT, blood test, etc.) | Then give me a checklist first |
|  |  | What is my disease?  (Leave the doctor's office after asking, go to the charge room (registration room) to ask how much the examination will cost, and then leave the hospital.) |
|  | The doctor requires non-intrusive examinations or tests and intrusive examinations or tests to be done together. | Discuss with the doctor first, "Can I do xx (non-intrusive examinations or tests) first, show you the results, and then do xx (intrusive examinations or tests)?" |
|  |  | After obtaining the doctor’s consent, do a non- intrusive examination or test and come back to see the doctor. |
|  |  | If the doctor disagrees, he will get the checklist and ask the doctor "What is my disease?"  (Leave the doctor's office after asking, go to the charge room (registration room) to ask how much the examination will cost, and then leave the hospital.) |
| **Intrusive treatment** | | |
|  | Let me give you an injection first, right? | Doctor, what medicine are you going to give me? |
|  | It is XXX medicine (or not provided) | (First write down the name and content of the medicine.)  Doctor, I will faint at the sight of a needle  (Leave after refusing the injection) |
|  | Infusion | Doctor, what medicine are you going to give me |
|  | The infusion is XXX medicine (or not provided) | (Write down the name and content of the medicine.)  I buy medicine first, but I still have something to do. I will come for the infusion later.  (Leave after refusing the infusion). |
|  | Hospitalized | I have something to do these days, and I will come back in a few days. |

# Appendix SC2 Implementation of Unannounced Standardized Patient Visit

Each doctor was visited by only one SP and each SP would only present one presumptive disease case. Five young females were recruited from local areas and they were intensively trained for two weeks to consistently and covertly present the presumptive asthma case to the providers in the sample. The SPs were randomly assigned to clinics and followed the normal procedures for any walk-in patient.

Upon presenting to the village doctor, SPs made an opening statement of the primary symptom(s) of the disease case. For this case, the SPs presented with a “Doctor, I have a shortness of breath; I am wheezing”. The SPs responded to all questions posed by the village doctor following a standardized script, purchased all medications prescribed (which are sold by village doctors in China) and paid providers any fee.

If doctors suggested any intrusive examinations, tests or treatment, patients will reject with a pre-determined excuse after asking doctors to specifying what it was. For example, if a village doctor showed an intention to give injections to SPs, SPs would reject by three steps. First, SPs will ask the village doctor what medicines they would like to prescribe for injection. Second, after a village doctor articulates the names of the medicines, SPs would then tell the doctors that “I will faint at the sight of a needle”. Third, if the doctors insisted on prescribing an injection, SPs would show their reluctance to have an injection and ask doctors to prescribe some oral medicines. If the doctor rejected to prescribe oral medicine and insisted on prescribing an injection, SPs would ask doctor what the disease she had and left the clinic.

# Appendix SC3 Questionnaire of Unannounced Standardized Patient Visit

**Note:**

1. Every morning, the enumerator should first check the standardized patient’s (SP) physical condition on that day (to ensure that the SP is healthy and does not show any symptoms, e.g. colds and fever), [Need to call XXX separately if the SP feels physically unwell.]
2. Before SP entering the clinic, the enumerator should help SP understand the number, photos, age, gender, and basic characteristics of village doctors in the clinic according to the Basic Information Sheet of Village Doctors in the manual. If there are multiple village doctors on the photo, the enumerator must clearly make sure which village doctor SP should visit.
3. After the SP walks out of the clinic, the enumerator and the SP should firstly fill in the first part of this questionnaire. And fill in the second part after finishing the audio recording.

| **Q0. The basic physical condition of the SP.**  [Note]: The enumerator should check the basic health condition of the SP before entering the clinic | | | |
| --- | --- | --- | --- |
|  | **Question** | **Option** | **Answer** |
|  | Did you cough on the day of the survey? | 1=Yes, 2=No |  |
|  | Did you have a fever on the day of the survey? | 1=Yes, 2=No |  |
|  | Did you feel a headache on the day of the survey? | 1=Yes, 2=No |  |
|  | Did you have a sore throat on the day of the survey? | 1=Yes, 2=No |  |
|  | Did you have a stuffy nose on the day of the survey? | 1=Yes, 2=No |  |
|  | Did you feel flustered on the day of the survey? | 1=Yes, 2=No |  |
|  | Did you have any other physical abnormal condition apart from symptoms above on the day of the survey? | 1= Yes, please describe  2= No |  |

| **Description of the consultation**  [Note]: please fill in the following parts according to the completion of the consultation | | | |
| --- | --- | --- | --- |
| **Question** | | **Option** | **Answer** |
|  | Was the village clinic a baseline sample village clinic or a new village clinic? | 1=Baseline sample village clinic  2=New village clinic **[Skip to Question 6]** |  |
|  | Did SP do consultation in this clinic at last? (See Q3 for the detail of consultation) | 1=Yes **[Skip to Question 4]**  2=No |  |
|  | What is the reason for not completing the consultation?  [Questionnaire END] | 1=The clinic was closed during the survey  2=The doctor identified the fake patient  3=Others, please indicate |  |
|  | Was the doctor who served the targeted doctor? | 1=Yes **[Skip to Question 8]**  2=No |  |
|  | What was the reason that the target doctor did not serve?  [Skip to Question 8] | 1=The target doctor clinic cannot back to the clinic during the survey  2=Other doctors in the clinic insisted to serve SP.  3=Others, please indicate |  |
|  | Was this clinic an alternative option？ | 1=Yes  2=No **[Skip to Question 8]** |  |
|  | The reason that change to the alternative clinic | 1=The target clinic is on the same street as the township health center  2=The target clinic only serve cases with Chinese medicine.  3=The target clinic was closed during the survey  4=Others, please indicate |  |
|  | Which version of case should be used? | 1=Version A  2=Version B |  |
|  | Was the version of SP actually used in this clinic the same as version that should be used? | 1=Same **[Skip to Q1]**  2=Different |  |
|  | If not, why? | 1=Because of SP  2=Because of the coordinator  3=Others, please indicate |  |

(1) After the visit, the enumerator interviews the SP and fills in.

| **Standardized patient reported information**  [Note]: After the SP patient finishes his medical treatment and walks out of the village clinic, the enumerator needs to check with the SP whether the village doctor who served was the village doctor that the SP should actually meet, according to age, gender, physical appearance and photo. | | | |
| --- | --- | --- | --- |
| **Question** | | **Option/unit** | **Answer** |
|  | What was the gender of the doctor who served you? | 1=Male 2=Female |  |
|  | What was the approximate age of the doctor who served you? | 1=Less than age 30  2=Age 30 - Age 39  3=Age 40 - Age 49  4=Age 50 - Age 59  5=Age 60 - Age 69  6=Above age 70 |  |
|  | The name of the doctor who served you (If you only know the last name, e.g. "Li", write "Dr Li". | Please indicate in words  If don’t know, write ‘999’ and skip to Question 9 |  |
|  | How did you know the name of the doctor?  **(Multiple choices. Please separate answers with ‘,’)** | 1=From the notice board outside  2=SP called himself or heard others call the doctor's name  3=From prescription  4=From badge  5=From table cards  6=Others, please indicate |  |
|  | Is the doctor on the SP report the doctor on the photo? | 1=Yes  2=No  3=No photo, could not identify  4=Photo was available but SP could not identify the doctor who served |  |
|  | Please describe the doctor's physical appearance and other characteristics  E.g.: was the doctor’s face or body different from other doctors on site, such as hair size and color; Wear glasses or not? Tall, short, fat, or thin? White or dark skin? | Please describe in words |  |
|  | The coordinator ensure whether the doctor for the SP report is the doctor who participated in the baseline survey (according to the village doctor’s basic information list in Annex 1 of the enumerator’s manual)? | 1=Yes  2=No |  |
|  | What is the code of the doctor's name in the SP report? | For doctors who have been surveyed, please look up from Annex 1 of the Enumerator’s Handbook; For doctors who have not been surveyed, fill in according to the coding rules |  |
|  | How many doctors were involved in the process? | 1=1 **[skip to Q2]**  2=More than 1 |  |
|  | Situation which multiple doctors are involved in the process of consultation | Please describe in words. |  |

| **Q2. Comprehensive assessment (SP tells its opinion on the doctor based on the situation of consultation)**  [Note]: This should be completed by SP itself, the coordinator should assist and ensure the SP fills in carefully. | | | |
| --- | --- | --- | --- |
| **Question** | | **Option/Unit** | **Answer** |
|  | In general, do you like this doctor? | 1=Strongly dislike 2= Slightly dislike  3=Neutral 4= Slightly like  5=Strongly like |  |
|  | If you want to see a doctor next time, do you want to see this doctor again? | 1=Yes  2=No |  |
|  | The doctor makes you feel relaxed, and you are willing to describe your symptoms and express your concerns to him. | 1=Strongly disagree 2= Slightly disagree  3=Neutral 4=Slightly agree  5=Strongly agree |  |
|  | To what extent that the doctor understands the disease? | 1=Very little understanding 2=Not quite understand  3=General 4= Quite understand  5=Very understand |  |
|  | In general, the doctor gave you adequate explanations and instructions during the consultation. | 1=Strongly disagree 2= Slightly disagree  3=Neutral 4=Slightly agree  5=Strongly agree |  |
|  | The doctor has fully explained and explained your treatment plan, including drug prescriptions, referrals, and orders. | 0=No treatment plan  1=Strongly disagree 2= Slightly disagree  3=Neutral 4=Slightly agree  5=Strongly agree |  |

| **Q3. Basic consultation situation**  [Note]: After confirming the basic situation of the village doctor that the SP visited, the enumerator and the SP should ensure the overall situation of the consultation, including the basic information asked by the village doctor, the situation of the treatment, and the suspicion to the SP, etc.. | | | |
| --- | --- | --- | --- |
| **Question** | | **Option/unit** | **Answer** |
|  | From the time you entered the clinic to the beginning of the consultation, how many patients (including the patients who was in consultation, excluding patients’ family members) were served by the doctor? | Person(s) |  |
|  | When you leave the clinic, how many patients are waiting to see the doctor (the least number of patients you think that were waiting for consultation, including those who were consulting, excluding patients’ family members)? | Person(s) |  |
|  | Did the doctor ask for your name? | 1=Yes 2=No |  |
|  | Did the doctor ask about your home address? | 1=Yes 2=No |  |
|  | Did the doctor ask the reason you came to this clinic? | 1=Yes 2=No |  |
|  | Under what circumstances did you end the consultation? | 1=After normal consultation **[Skip to Question 8]**  2=The doctor refused to serve the patient (did not conduct any consultation) after the doctor listened to the opening remarks,  3=The doctor referred directly after listening to the opening remarks, without any inquiries (or just asked about the content of the opening remarks)  4=Because of refusal to do **invasive examination**, I stopped the consultation and left. **[Skip to Question 8]**  5=Because of refusal to accept **injections or infusions**, I stopped the consultation and left. **[Skip to Question 8]**  6=Because of refusal to accept **other on-site treatments**, I stopped the consultation and left. **[Skip to Question 8]**  7=The doctor suspects that I was a fake patient **[Skip to Question 9]**  8=Others, please specify **[Skip to Question 8]** |  |
| 7. | Reason(s) for the doctor's refusal | 1=The doctor did not serve such patients  2=Other, please specify |  |
| 8. | Do you think the doctor suspected that you were a fake patient? | 1=Yes  2=No **[Skip to Q4]** |  |
| 9. | If so, what is the situation? | Please describe in words |  |

| **Q4. Medical tests (the enumerator needs to reconfirm according to the record)**  [note]:  1. The enumerator should ask the SP for all the recommended tests and fill out this form.  2. After completing the text version of the recording, reconfirm that all the tests suggested by the doctor are correct and included. | | | |
| --- | --- | --- | --- |
| **Question** | | **Option/unit** | **Answer** |
|  | Does the doctor ask you to do or suggest you do any tests? | 1=Yes 2=No **[Skip to Q5]** |  |
|  | Pulse | 1=Yes 2=No |  |
|  | Blood pressure | 1=Yes 2=No |  |
|  | Chest auscultation | 1=Yes 2=No |  |
|  | Back auscultation | 1=Yes 2=No |  |
|  | Body temperature (thermometer) | 1=Yes 2=No |  |
|  | Body temperature (hand touching) | 1=Yes 2=No |  |
|  | Check the throat or its surrounding area | 1=Yes 2=No |  |
|  | Pulmonary ventilation function test | 0=No  1=It is recommended to do it in this clinic and did it  2=It is recommended to do it in this clinic, but not done  3=It is recommended to go to the township health centre for inspection  4=It is recommended to go to hospitals at county level and above for the test |  |
|  | Bronchial dilation test | 0=No  1=It is recommended to do it in this clinic and did it  2=It is recommended to do it in this clinic, but not done  3=It is recommended to go to the township health centre for inspection  4=It is recommended to go to hospitals at county level and above for the test |  |
|  | Bronchial provocation test | 0=No  1=It is recommended to do it in this clinic and did it  2=It is recommended to do it in this clinic, but not done  3=It is recommended to go to the township health centre for inspection  4=It is recommended to go to hospitals at county level and above for the test |  |
|  | Bronchial test (the doctor did not specify what kind of bronchial examination, but mentioned that the bronchus should be checked) | 0=No  1=It is recommended to do it in this clinic and did it  2=It is recommended to do it in this clinic, but not done  3=It is recommended to go to the township health centre for inspection  4=It is recommended to go to hospitals at county level and above for the test |  |
|  | Are there any other tests that the doctor required or recommended you do? | 1=Yes 2=No **[Skip to Question 22]** |  |
|  | Test one | Please describe the name of the test in word. |  |
|  | Where does the doctor recommended you go for the above test (test one)? | 0=No  1=It is recommended to do it in this clinic and did it  2=It is recommended to do it in this clinic, but not done  3=It is recommended to go to the township health centre for inspection  4=It is recommended to go to hospitals at county level and above for the test |  |
|  | Test two | Please describe the name of the test in word. |  |
|  | Where does the doctor recommended you go for the above test (test two)? | 0=No  1=It is recommended to do it in this clinic and did it  2=It is recommended to do it in this clinic, but not done  3=It is recommended to go to the township health centre for inspection  4=It is recommended to go to hospitals at county level and above for the test |  |
|  | Test three | Please describe the name of the test in word. |  |
|  | Where does the doctor recommended you go for the above test (test three)? | 0=No  1=It is recommended to do it in this clinic and did it  2=It is recommended to do it in this clinic, but not done  3=It is recommended to go to the township health centre for inspection  4=It is recommended to go to hospitals at county level and above for the test |  |
|  | Test four | Please describe the name of the test in word. |  |
|  | Where does the doctor recommended you go for the above test (test four)? | 0=No  1=It is recommended to do it in this clinic and did it  2=It is recommended to do it in this clinic, but not done  3=It is recommended to go to the township health centre for inspection  4=It is recommended to go to hospitals at county level and above for the test |  |
|  | Is there any harmful test for all the above-mentioned tests done in the clinic? | 1=Yes 2=No **[Skip to Q5]** |  |
|  | If so, does the SP refuse to conduct harmful inspections in accordance with the principle? | 1=Yes **[Skip to Q5]**  2=No |  |
|  | If the principles are not followed, what is(are) the reason(s) the SP reject harmful inspections? | Please describe the name of the test in word. |  |

| **Q5. Costs (write ‘0’if no)**  [Note]: The actual cost of the SP's consultation in the village clinic, write ‘0’ if there was no. | | | |
| --- | --- | --- | --- |
| **Question** | | **Option/unit** | **Answer** |
|  | Actual total cost of the consultation | Yuan **[skip to Q6 if ‘0’]** |  |
|  | Can the total cost distinguish between the medical expenses and the drug expenses? | 1=Yes  2=No **[Skip to Q6]** |  |
|  | General medical expenses-basic expenses | 元  Yuan |  |
|  | General Medical expenses-injection fees (including injections, infusions, boluses, etc.) | 元  Yuan |  |
|  | Tests expenses | 元  Yuan |  |
|  | Drug expenses | 元  Yuan |  |

The enumerator fills in the part based on the record

**Note:**

1) After the enumerator and the SP have prepared the transcript of the recording together, fill in the following content based on the transcript. Check this part of the content based on the proofread transcript the next day.

2) First, fill in the symptoms and condition inquiries described by SP in Q6, as well as the diagnosis results and treatment plan. According to the doctor's questioning behavior in the proofread transcript

3) Finally, calculate the SP’s time to enter and leave the village clinic in Q9, as well as the length of time for each doctor’s questioning behavior during the consultation.

| **Q6. Symptoms and condition inquiry**  [Note]:  1). Organise the text of the recording, according to the consultation information of the village doctor,  2). Judging the questions raised by the village doctor during the consultation and check whether the SP mentioned the answer to the question according to the transcript. | | | |
| --- | --- | --- | --- |
| **Question** | | **Did the doctor ask the following questions?**  1=Yes **[Move to next row]**  2=No | **Did you tell the doctor the information in the answer to this question?**  1=Yes 2=No |
|  | When did you gasp recently? Are you gasping now?  How many days has it been? |  |  |
|  | When did you gasp last night? What time did you gasp? |  |  |
|  | What did you do when you were gasping last night? What did you do yesterday? |  |  |
|  | How long did you gasp last night? |  |  |
|  | Was it a bad gasp last night? |  |  |
|  | What was the difference between the gasp last night and before?  Was it more serious last night? |  |  |
|  | How did you recover last night? |  |  |
|  | Did you clean normally or clean the old stuffs (lots of dusts) last night? Did you gasp after cleaning or within cleaning? |  |  |
|  | When did it start? |  |  |
|  | How long has it been so breathless? How many days has it been? Was there such a condition before? |  |  |
|  | Was the period of gasp from a year ago to two or three months ago? |  |  |
|  | How often does the breathless condition occur? When did the last one happens before yesterday? |  |  |
|  | Has it changed in recent compared to what it was before? Has it been serious recently? |  |  |
|  | How long did you gasp before? |  |  |
|  | Was your gasp serious before? |  |  |
|  | Is it difficult to breathe when gasping? |  |  |
|  | Is there a special sound when breathing? Is there a sound in your throat? |  |  |
|  | When gasping, did you feel hard to breathe out or breathe in? |  |  |
|  | Did you cough? |  |  |
|  | How long is the cough? |  |  |
|  | Did you cough or gasp first? |  |  |
|  | Is the cough bad? |  |  |
|  | Did you sputum? |  |  |
|  | Did you cough up blood? |  |  |
|  | Did you have sore throat? |  |  |
|  | Was your throat dry? |  |  |
|  | Is this situation worse in the morning or at night? When is the day more serious? |  |  |
|  | Is it easier to happen in winter? |  |  |
|  | Did you gasp during the day? |  |  |
|  | Did you gasp normally? Did you often out of breath? |  |  |
|  | Did you gasp before or after sleeping? |  |  |
|  | Is there any symptom of upper respiratory tract infection (cold, sneezing, blocked nose)? |  |  |
|  | Under what circumstances did it happen before? What were you doing every time it happens? |  |  |
|  | Can you stay in a dusty environment? |  |  |
|  | 对什么东西过敏吗？  Are you allergic to anything? |  |  |
|  | Is there any smell that cause allergies? |  |  |
|  | Is there any pollen allergy? |  |  |
|  | Will there be onsets/allergies from contact with animals? |  |  |
|  | How does the symptom disappear/relieve/improve? |  |  |
|  | Have you seen a doctor before? |  |  |
|  | Why didn't you see a doctor before, but do this time? Is it more serious this time? |  |  |
|  | Do you feel tired/exhaust/lack of energy? |  |  |
|  | Any lung illness? |  |  |
|  | Do you have fever? |  |  |
|  | Do you have chest pain? |  |  |
|  | Do you feel flustered? |  |  |
|  | Has the weight changed? Are you thinner? Did you lose weight? |  |  |
|  | Do you sweat at night (sweat a lot when you fall asleep)? |  |  |
|  | Do you have stomachache? |  |  |
|  | What do you usually eat? |  |  |
|  | Do you smoke? |  |  |
|  | Do you drink? |  |  |
|  | Did you have this symptom when you were a child? |  |  |
|  | Does anyone in the family have the same symptom? |  |  |
|  | Has your older brother visited the hospital? |  |  |
|  | What is your older brother's disease? |  |  |
|  | Does your older brother take medicine? |  |  |
|  | What medicine did your older brother take? |  |  |
|  | Apart from your older brother, is there anyone else in the family who has the same symptom? |  |  |
|  | Name |  |  |
|  | Do you have health insurance? |  |  |
|  | What is your family’s financial situation? |  |  |
|  | Are you caught in the rain？ |  |  |
|  | When is your date of menstruation？ |  |  |
|  | What is your job? |  |  |
|  | Why come here to see a doctor? |  |  |
|  | What are you doing here? Why do you come here? |  |  |
|  | Where is you friend’s home? |  |  |
|  | Is your friend's home in our village? Where is your friend's home in detail? |  |  |
|  | What is your friend’s name? |  |  |
|  | Have you taken any medicine? |  |  |
|  | What medicine/ anti-inflammatory did you take? |  |  |
|  | When did you take medicine? |  |  |
|  | Has the symptom improved after taking medicine? |  |  |
|  | How did you to take? How much did you take? |  |  |
|  | Where did you buy the medicine? When did you buy the medicine? |  |  |
|  | Did you take any other medicines? |  |  |
|  | Age |  |  |
|  | Family address |  |  |

| **Q7.** **Diagnosis and treatment plan** | | | | |
| --- | --- | --- | --- | --- |
| **Question** | | **Option/unit** | **Answer** | |
|  | Did the doctor tell SP the diagnosis result? | 1=The doctor took the initiative to tell  2=The doctor did not take the initiative to tell but the patient asked  3=The doctor did not take the initiative to tell and the patient forgot to ask **[skip to question 3]**  4=The doctor did not take the initiative to tell, the patient asked, but the doctor still did not tell **[skip to question 3]** |  | |
|  | **What is the diagnosis given by the doctor?**  (Please use words to explain, in accordance with the order in which the doctor gave the diagnosis, list all the diagnosis results that the doctor gave to the SP.)  If the doctor emphasises a certain diagnosis result, this item should be written first, and the others are listed in the original order.  E.g: the village doctor said that it may be a or b, so write a first and then b. If the doctor emphasized that b has larger probability, write b first and then a. | List serial number and line break in order, write like:  1. Cold  2. Lung infection) |  | |
|  | Does the doctor recommend a referral? (the referral also includes the doctor’s recommendation to go to other hospitals for test) | 1=Yes  2=No **[Skip to Question 6]** |  | |
|  | Did the doctor explicitly recommend immediate referral? | 1=Yes 2=No |  | |
|  | If a referral is recommended, please write down the level of the institution where the referral is recommended | 1=Other village clinics  2=Township Health Center  3=County hospital  4=Municipal hospital  5=Other, please specify |  | |
|  | Did the doctor ask to come back for follow-up? (If yes, in which of the following A-E situations? A-E are multiple choices) | 1=Yes  2=No **[Skip to Question 12]** |  | |
|  | Situation A: I feel that my condition has not improved | 1=Yes 2=No |  | |
|  | Situation B: Buy medicine again after finishing the given medicine | 1=Yes 2=No |  | |
|  | Situation C: Completed the test in other institutions requested by the doctor. | 1=Yes 2=No |  | |
|  | Situation D: Worsening condition (some serious symptoms appear) | 1=Yes 2=No |  | |
|  | Situation E: Others, please specify | 1=Yes 2=No |  | |
|  | Did the doctor request on-site treatment? (Such as plasters, oxygen inhalation, sprays, acupuncture and massage, cupping, baking lamps (magic lamps), etc. | 1=Yes  2=No **[Skip to Question 17]** |  |  |
|  | If on-site treatment was required, what is it? | Please describe in words |  |  |
|  | If **on-site treatment** was required, does SP refuse **on-site treatment** in principle? | 1=Reject once in principle  **[Skip to question 16]**  2=Reject twice according to the principle  **[Skip to question 16]**  3=No |  |  |
|  | If the principles were not followed, on what grounds does SP refuse **on-site treatment**? | Please describe in words |  |  |
|  | If **on-site treatment** was required, what did the doctor do in the end? | 1=The doctor prescribed oral medications  2=The doctor insisted on requesting **on-site treatment**, SP refused and left the clinic.  3=The doctor allowed SP’s on-site treatment of medicines or equipment to be bought back first, SP purchased and ended the medical treatment.  4=Other, please specify |  |  |
|  | Did the doctor recommend any aerosol (nebulized spray)? | 1=Yes, 2=No |  |  |
|  | Did the doctor give medical advice (in addition to taking medicine)? | 1=Yes  2=No **[Skip to Q8]** |  |  |
|  | Did the doctor have any suggestions regarding diet? | 1=Yes, 2=No |  |  |
|  | Did the doctor have any suggestions regarding drinking? | 1=Yes, 2=No |  |  |
|  | Did the doctor have any advice on mentality? | 1=Yes, 2=No |  |  |
|  | Did the doctor have any advice on staying away from air pollution (dust)? | 1=Yes, 2=No |  |  |
|  | Did the doctor have any advice on home hygiene? | 1=Yes, 2=No |  |  |
|  | Did the doctor ask the patient to be hospitalized? | 1=Yes, 2=No |  |  |

| **Q8. Drug records (decimals are allowed)**  [Note]: After the SP ends, if the doctor prescribed medicine to the patient, please fill in the medicine form and complete the following questions. If not, just fill in Question 1. | | | |
| --- | --- | --- | --- |
| **Question** | | **选项/单位**  **Option/unit** | **Answer** |
|  | Did the village doctor prescribe medicine to the patient? (Prescribed medicines also include medicines that was mentioned but not available in the clinic, and medicines that the doctor wants to use in infusions or bolus injections) | 1=Yes  2=No **[Skip to Q9]** |  |
|  | Did the doctor prescribe Chinese patent medicines (Chinese patent medicines are Chinese medicines with fixed packaging and specifications)? | 1=Yes  2=No  3= In small paper bag, could not identify |  |
|  | Did the doctor want to prescribe Chinese herbal medicine? | 1=Yes  2=No **[Skip to Question 7]** |  |
|  | If so, did the SP refuse to prescribe Chinese herbal medicines in principle? | 1=Reject once in principle **[Skip to Question 6]**  2=Reject twice in the principle **[Skip to Question 6]**  3=No |  |
|  | If the principle was not followed, in what reason did SP refuse to let the doctor prescribe Chinese herbal medicine? | Please describe in words |  |
|  | If so, what did the doctor do in the end? | 1=The doctor prescribed oral medications  2=The doctor insisted on prescribing Chinese herbal medicine, SP refused and left the clinic.  3=Other, please specify |  |
|  | Did the doctor require infusion or intramuscular injections? | 0=No [Skip to the drug list]  1=Require infusion  2=Requires intramuscular injection **[Skip to Question 13]** |  |
|  | If an **infusion** was required, what medicine the doctor planned to use? (If SP asked but the doctor did not say, write ‘999’; if SP forgot to ask, write ‘888’) | Please describe in words |  |
|  | If an **infusion** was required, does the SP refuse the **infusion** in principle? | 1=Reject once in principle **[Skip to Question 11]**  2=Reject twice in the principle **[Skip to Question 11]**  3=No |  |
|  | If the principle was not followed, what reason does SP refuse the **infusion**? | Please describe in word |  |
|  | If an **infusion** was required, what did the doctor do in the end? | 1=The doctor prescribed oral medications  2= The doctor insisted on-site infusion, SP refused and left the clinic.  3=Other, please specify |  |
|  | Did the doctor ask for an **intramuscular injection**? | 1=Yes  2=No **[Skip to the Drug list]** |  |
|  | If an **intramuscular injection** was required, what medicine the doctor planned to use?  (If SP asked but the doctor did not say, write "999"; if SP forgot to ask, write "888") | 请用文字说明  Please describe in word |  |
|  | If there was a request for an **intramuscular injection**, did the SP refuse to receive an **intramuscular injection** in principle? | 1=Reject once in principle **[Skip to Question 16]**  2=Reject twice in the principle **[Skip to Question 16]**  3=No |  |
|  | If the principle was not followed, in what reason did SP refuse to take **intramuscular injection?** | Please describe in word |  |
|  | If an **intramuscular injection** was required, what did the doctor do in the end? | 1=The doctor prescribed oral medications  2=The doctor insisted on getting **on-site** intramuscular injection, SP refused and left the clinic.  3=Other, please specify |  |

[Note]: Fill out the following drug list based on the recording transcript, SP medical prescription and purchased drugs.

1. The medicine should be taken based on the doctor's prescription. If the medicine specifications on the prescription are different from the actual medicine, fill in the information which was notified by the doctor. If the doctor did not tell how to take it, fill in the information on the medicine.

2. If the medicine prescribed by the doctor is wrapped in a small paper bag and mixed together, the name of the medicine is written "small medicine bag", only 12-15 questions need to be filled in.

3. Case a: The doctor prescribed one bottle of Compound Licorice Tablets (Fufang Gancao Pian), each bottle contains 100 tablets, each tablet contains 112.5 mg of licorice extract powder, take orally three tablets at each time, twice a day, six days in total, no need to suspend taking.

4. Case b: The doctor prescribed six bags of Ribavirin Granules (Liba Weilin Keli); 100mg per bag, orally taken, three times a day, 1 bag per time, no need to suspend taking.

5. Case c: The doctor prescribed 20 tablets of **a kind of** tablet drug, fill in ‘unknown drug’ on drug name, unknown dosage, two tablets per time, two times per day, five days in total, no need to suspend taking.

| **No.** | **1. Drug name**  **(No need for brand name)** | **2. Dosage form**  1=Tablet  2=Capsule  3=Mixture (including oral liquid)  4=Powder  5=Pill  6=Granule  7=Injection  8=Power injection  9=Liquid injection  10=Syrup  11=Patch  12=Gel  13=Ointment  14=Spray  15=Others, please indicate | **3. How to take product**  1=Oral  2=Intravenous drip  3=Intravenous bolus  4=Subcutaneous injection  5=Intramuscular injection  6= Patch  7=Enema  8=Spray  **9=**Others, please indicate | **4. Is this drug available in the clinic?**  1=Yes  **2=**No》skip to next drug | **5. Type of packing**  1=Box  2=Bottle  3=Bag  4=Board  5=Tablet  6=Tube  7=Granule  8=Pill  9=Mixed in paper bag. (**Ignore Questions 8-12 if option 9 is chosen here**.)  10=Others, please indicate | **6. The quantity that the doctor prescribed**  (the unit should correspond to the question No.5) | **7. Within the smallest unit packaging type, the smallest divisible packaging type**  1=Box  2=Bottle  3=Bag  4=Board  5=Tablet  6=Tube  7=Granule  8=Pill  9=Mixed in paper bag. (**Ignore Questions 8-12 if option 9 is chosen here**.)  10= Others, please indicate | **8.** **Within the smallest unit packaging type, the smallest divisible specification and quantity**  (correspond to Question 7) | **9. Within the smallest unit packaging type, the smallest divisible dosage unit**  1=g  2=mg  3=ml  4=IU  5=Others, please indicate | **10. Within the smallest divisible dosage unit, what is the dosage of the drug?**  (If there is no specification, fill in the content of the main ingredient where the unit should correspond to Question 9. For compound preparations, please note **‘Main ingredient specification’**. If no specification, write ‘888’) | **11. National drug approval code (if there is no code, write ‘888’)** | **12.Dosage per use**  **[correspond to Question 7]** | **13. How many times to use per day?**  time/day | **14. How many days in total?**  Day(s) | **15.Whether need to suspend taking the drug during treatment?**  1=Yes  2=No |
| --- | --- | --- | --- | --- | --- | --- | --- | --- | --- | --- | --- | --- | --- | --- | --- |
| 1. **a** | Compound Licorice Tablets | **1** | **1** | **1** | **2** | **1** | **5** | **100** | **2** | **112.5** | **H44020984** | **3** | **2** | **6** | **2** |
| 1. **b** | Ribavirin Granules | **6** | **1** | **1** | **3** | **6** | **3** | **1** | **2** | **100** | **H51023509** | **1** | **3** | **2** | **2** |
| 1. **c** | Unknown drug | **1** | **1** | **1** | **5** | **20** | **5** | **1** | **5** | **888** | **888** | **2** | **2** | **5** | **2** |

| **Q9. Consultation length**  【Note】:  1. Unified conversion to the length of time in seconds.  2. The recording time of Questions 1-2 is the recording time of entering and leaving the clinic.  3. Questions 3-9, calculate the length of time spent, **the total time length of** **Questions 3-9 must be equal to the length of time obtained from Question 2 minus question 1. If there may be alternation between behaviors, estimate the time.**  **4. Questions 4-5, if the doctor asked a doctor while checking, divide this period of time into two parts.**  5. For the prescription time in Question 6, if the doctor's prescription time was separated, need to sum up these times together. In addition, the treatment plan includes referral, follow-up, hospitalization, and doctor's order. | |
| --- | --- |
| 1. Point of time of entering the village clinic **(seconds)** |  |
| 1. **Point of time** of leaving the village clinic **(seconds)** |  |
| 1. **The length of time (seconds)** between entering the village clinic and the start doctor's consultation |  |
| 1. **The length of time** for consultation **(seconds)** |  |
| 1. **The length of time** for test (including walking and waiting time) **(seconds)** |  |
| The length of time to give the diagnosis result (seconds) |  |
| 1. The length of time to give the **treatment plan (including the time to write a prescription or orally tell the treatment plan) (seconds)** |  |
| 1. **The length of time** to take the medicine **(excluding the prescription time, starting from the action of taking the medicine)** (seconds) |  |
| 1. **The length of time** from the last conversation with the village doctor to leaving the village clinic (seconds) |  |
| 1. From the beginning of the consultation to the end of the consultation, the length of time (seconds) that the consultation process was interrupted or waited due to other people or things |  |
| 1. From the beginning of the consultation to the end of the consultation, the length of time (seconds) that the consultation process was interrupted or waited for due to SP personal reasons |  |

| **Q10. Recording**  [Note]: After finishing the recording transcript and filling in the other parts of the questionnaire, complete this part. | | | |
| --- | --- | --- | --- |
| **Question** | | **Option/unit** | **Answer** |
|  | How was the recording quality of this consultation? | 1=The recording was complete and clear **[End questionnaire]**  2=The recording was complete but cannot be heard clearly  3=The recording was incomplete but very clear  4=The recording was incomplete and unclear  5=Loss of recording  6=Other, please specify |  |
|  | If there was a problem with the recording, what was the specific reason(s)?  (Multiple choice) | 1=Mobile phone issue (power failure, incoming call, etc.)  2=SP turned off the phone recording  3=During the recording, the phone was far away from the doctor  4=During the recording, other sounds was too loud  5=The recording was deleted during the recording copy process  6=Other, please specify |  |

# Appendix SD Implementation of Clinical Vignette

Each vignette was administered by two enumerators who were trained by the research team in classroom and in the field for seven days before to present the standardized clinical vignette to VCs. The enumerators were provided a script, which included disease symptoms, history, and background story, before and during the vignette presentation. One enumerator played the role of the ‘*mock patient’*; the other enumerator assumed the role of ‘*the facilitator*’ to read the instructions to the village doctor, document the interaction between doctor and mock patient, and provided additional information that the patient might not know but would be helpful to the doctor and his/her diagnosis if he/she actively solicited it, *e.g.*, the results of tests or examinations.

To begin the vignette, the facilitator informed the village doctor that a young woman was visiting the clinic. The ‘*mock patient*’ then told the doctor about her problem with an opening statement — “Doctor, I have a shortness of breath; I am wheezing”—the primary symptom(s) of the disease case. The village doctor was then asked to proceed as if with a real patient and were told that the patient would answer any questions asked and comply with any given instructions. If the village doctor did not provide any diagnosis, the ‘*mock patient*’ was instructed to ask for a diagnosis at the end of the interaction. During the interaction, the ‘*facilitator*’ documented the village doctor’s questions, diagnostic examination and tests requested, stated diagnosis, treatment prescribed (drugs or advices), and potential patient referral(s).

For each drug that village doctors prescribed, the village doctor was asked about the dosage form, route of administration, and availability of the drug in the clinic at the end of the clinical vignette. If the medicine is immediately available in the clinic, we further asked village doctors to provide information on dosing regimen, frequency of drug administration, and number of days with medication.

# Appendix SE Standards for Classification of Prescribed Drugs

**Correct medicines** include inhaled corticosteroids, oral corticosteroids, long-acting β receptor agonists, short-acting β receptor agonists, leukotriene inhibitors, theophylline, bronchodilator, inhaled anticholinergics, or cromones.

**Potentially harmful medicines** include the drugs possibly making asthma worse such as aspirin, β-blockers, etc.

**Unnecessary medicines** are those that are not on the list of correct and potential harmful medicines.

# Appendix Table S1 Comparison of Characteristics of Providers and Clinics (by Prescription Behavior in Vignette and Standard Patient Visit)

| Variable | Doctors who prescribed only in Vignettes | Doctors who prescribed only in SPs | Difference  (95% confidence level) |
| --- | --- | --- | --- |
| Number of observations | 17 | 31 |  |
| Clinician characteristics |  |  |  |
| Male (yes = 1) | 64.71 (11/17) | 58.06 (18/31) | -6.64  (-36.13, 22.85) |
| Age (year) | 45.82 (10.57) | 42.19 (6.12) | -3.63  (-8.34, 1.08) |
| Junior high school or lower education (yes = 1) | 5.88 (1/17) | 3.23 (1/31) | -2.66  (-14.71, 9.39) |
| Senior high school (yes = 1) | 82.35 (14/17) | 67.74 (21/31) | -14.61  (-41.13, 11.91) |
| Junior college or higher education (yes = 1) | 11.76 (2/17) | 29.03 (9/31) | 17.27  (-7.63, 42.17) |
| (Associate) Medical practitioner (yes = 1) | 11.76 (2/17) | 12.90 (4/31) | 1.14  (-18.84, 21.12) |
| Familiarity with the term “clinical pathway” (yes = 1) | 0.00 (0/17) | 32.26 (10/31) | 32.26**  (9.56, 54.96) |
| Received any training on asthma in the previous two years (yes = 1) | 0.00 (0/17) | 0.00 (0/31) | -- |
| Received any training on the use of antibiotics in the previous two years (yes = 1) | 47.06 (8/17) | 61.29 (19/31) | 14.23  (-15.46, 43.92) |
| Income from working in clinic (%) | 88.30 (24.44) | 96.16 (10.05) | 7.87  (-1.92, 17.65) |
| Clinic characteristics |  |  |  |
| Number of village providers (persons) | 1.88 (1.11) | 2.26 (1.06) | 0.38  (-0.26, 1.01) |
| Number of patient visits in the previous month (encounters) | 232.82 (266.49) | 343.35 (536.09) | 110.53  (-161.91, 382.97) |
| Implementation of zero-mark-up drug policy (yes = 1) | 100.00 (17/17) | 96.77 (30/31) | -3.23  (-11.81, 5.35) |
| Varieties of Western drugs in stock | 61.71 (30.30) | 79.86 (60.07) | 18.16  (-12.55, 48.86) |
| Varieties of Chinese patent medicines in stock | 25.59 (22.09) | 32.24 (23.48) | 6.65  (-7.11, 20.42) |
| Availability of medical instruments/services that may be used for the diagnosis of asthma ^a^ |  |  |  |
| Instruments for chest auscultation (yes = 1) | 100.00 (17/17) | 96.77 (30/31) | -3.23  (-11.81, 5.35) |
| Instruments for pulmonary ventilation test (yes = 1) | 0.00 (0/17) | 0.00 (0/31) | -- |
| Instruments for bronchodilator test (yes = 1) | 0.00 (0/17) | 0.00 (0/31) | -- |
| Instruments for assessment of heart rate, pulse, or blood pressure (yes = 1) | 100.00 (17/17) | 100.00 (31/31) | -- |
| Instruments for chest X-ray examination (yes = 1) | 5.88 (1/17) | 0.00 (0/31) | -5.88  (-14.34, 2.58) |
| Instruments for blood routine test (yes = 1) | 23.53 (4/17) | 3.23 (1/31) | -20.30*  (-37.80, -2.80) |
| Instruments for percussion added to chest examination (yes = 1) | 100.00 (17/17) | 100.00 (31/31) | -- |
| Availability of intravenous drip (yes=1) | 100.00 (17/17) | 93.55 (29/31) | -6.45  (-18.38, 5.48) |
| Availability of intramuscular injection (yes = 1) | 100.00 (17/17) | 90.32 (28/31) | -9.68  (-24.03, 4.68) |
| Keeps medical records (yes = 1) | 94.12 (16/17) | 90.32 (28/31) | -3.80  (-20.46, 12.87) |
| Incentives from upper-levels tied to prescription assessment (yes = 1) | 94.12 (16/17) | 80.65 (25/31) | -13.47  (-34.44, 7.50) |

**Note:** a. According to standards stated in the Chinese national practice guidelines to treat asthma, seven examinations and/or tests are recommended for the diagnosis of asthma.

# Appendix Table S2 Prescriptions in the Treatment of a Presumptive Asthma

| Variable | *Know* | *Can* | *Do* | *Know-Can* Gap | *Can-Do* Gap |
| --- | --- | --- | --- | --- | --- |
| Number of encounters | 370 | 370 | 104 | 740 | 474 |
| 1. Encounters with prescriptions (yes = 1) | 54.32 (201/370) | 50.54 (187/370) | 67.31 (70/104) | 3.78  (-3.42, 10.98) | -16.77**  (-27.52, -6.01) |
| 2. Encounters in which prescribed medicines were loose ^a^ (yes = 1) | NA | NA | 5.77 (6/104) | NA | NA |
| Encounters assessed | 201 | 187 | 64 | 388 | 251 |
| 3. Encounters in which only Chinese patent medicines were prescribed (yes = 1) | 10.95 (22/201) | 11.76 (22/187) | 35.94 (23/64) | -0.82  (-7.15, 5.51) | -24.17***  (-34.68, -13.66) |
| 4. Encounters in which only Western medicines were prescribed (yes = 1) | 48.26 (97/201) | 53.48 (100/187) | 23.44 (15/64) | -5.22  (-15.19, 4.75) | 30.04***  (16.34, 43.74) |
| 5. Encounters in which both Chinese patent medicines and Western medicines were prescribed (yes = 1) | 40.80 (82/201) | 34.76 (65/187) | 40.63 (26/64) | 6.04  (-3.63, 15.70) | -5.87  (-19.55, 7.82) |
| 6. Number of medicines prescribed per encounter | 4.17 (2.47) | 3.67 (2.39) | 2.63 (1.28) | 0.51*  (0.02, 0.99) | 1.04***  (0.43, 1.66) |
| 7. Number of medicines prescribed via injection per encounter | 2.43 (2.73) | 2.22 (2.53) | NA | 0.21  (-0.32, 0.73) | NA |
| 8. Number of non-injection medicines per encounter | 1.75 (1.36) | 1.45 (1.25) | 2.63 (1.28) | 0.30*  (0.04, 0.56) | -1.18***  (-1.53, -0.82) |
| 9. Number of Chinese patent medicines prescribed per encounter | 0.76 (0.89) | 0.64 (0.80) | 1.22 (0.95) | 0.12  (-0.05, 0.29) | -0.58***  (-0.82, -0.34) |

**Note:** a. When providers provided loose medications (medications taken from original packaging and did not give a written or oral prescription) in the standardized patient (SP) visits, the exact name of these medicines and related information is undeterminable. Six encounters with loose medicines were excluded from the rest of the analysis.

*p*>0.05, * *p*≤0.05, ** *p*<0.01, *** *p*<0.001

# Appendix Table S3 Irrational Use of Medicine in the Treatment of a Presumptive Asthma

| Variable | *Know* | *Can* | *Do* | Total Gap | *Know* Gap | *Know-Can* Gap | *Can-Do* Gap |
| --- | --- | --- | --- | --- | --- | --- | --- |
| Number of encounters assessed ^a^ | 179 | 165 | 41 | 41 | 179 | 344 | 206 |
| (1) Only correct medicines prescribed | 9.50 (17/179) | 7.88 (13/165) | 12.20 (5/41) | 87.80***  (77.66, 97.95) | 90.50***  (86.20, 94.81) | 1.62  (-4.36, 7.60) | -4.32  (-14.00, 5.37) |
| (2) Only unnecessary medicines prescribed | 37.99 (68/179) | 50.91 (84/165) | 75.61 (31/41) | -75.61***  (-88.92, -62.30) | -37.99***  (-45.12, -30.86) | -12.92*  (-23.37, -2.47) | -24.70**  (-41.43, -7.97) |
| (3) Both correct and unnecessary medicines prescribed | 52.51 (94/179) | 41.21 (68/165) | 12.20 (5/41) | -12.20*  (-22.34, -2.05) | -52.51***  (-59.85, -45.18) | 11.30*  (0.78, 21.82) | 29.02***  (13.07, 44.97) |
| (4) Only potentially harmful medicines prescribed | 0.00 (0/179) | 0.00 (0/165) | 0.00 (0/41) | -- | -- | -- | -- |
| (5) Both correct and potentially harmful medicines prescribed | 0.00 (0/179) | 0.00 (0/165) | 0.00 (0/41) | -- | -- | -- | -- |
| (6) Unnecessary and potentially harmful medicines prescribed | 0.00 (0/179) | 0.00 (0/165) | 0.00 (0/41) | -- | -- | -- | -- |
| (7) Correct, unnecessary, and potentially harmful medicines prescribed together | 0.00 (0/179) | 0.00 (0/165) | 0.00 (0/41) | -- | -- | -- | -- |
| Encounters with any of the following irrational uses of medicines | 77.09 (138/179) | 85.45 (141/165) | 65.85 (27/41) | -65.85***  (-80.55, -51.16) | -77.09***  (-83.27, -70.92) | -8.36*  (-16.62, -0.10) | 19.60**  (6.54, 32.66) |
| (1) Antibiotics prescribed | 64.80 (116/179) | 62.42 (103/165) | 63.41 (26/41) | -63.41***  (-78.34, -48.49) | -64.80***  (-71.82, -57.79) | 2.38  (-7.82, 12.58) | -0.99  (-17.62, 15.64) |
| (2) Injections prescribed | 55.31 (99/179) | 57.58 (95/165) | 0.00 (0/41) | 0.00  (0.00, 0.00) | -55.31***  (-62.61, -48.00) | -2.27  (-12.79, 8.25) | 57.58***  (42.37, 72.78) |
| (3) Polypharmacy (> 5 drugs) | 37.99 (68/179) | 32.12 (53/165) | 0.00 (0/41) | 0.00  (0.00, 0.00) | -37.99***  (-45.12, -30.86) | 5.87  (-4.24, 15.98) | 32.12***  (17.76, 46.48) |
| (4) Incompatible medicines prescribed | 10.06 (18/179) | 10.30 (17/165) | 0.00 (0/41) | 0.00  (0.00, 0.00) | -10.06***  (-14.47, -5.64) | -0.25  (-6.66, 6.17) | 10.30*  (0.95, 19.65) |
| (5) Repetitive medicines prescribed | 8.38 (15/179) | 6.67 (11/165) | 4.88 (2/41) | -4.88  (-11.55, 1.80) | -8.38***  (-12.45, -4.31) | 1.71  (-3.89, 7.32) | 1.79  (-6.56, 10.14) |
| (6) Medicines prescribed having adverse interactions | 7.82 (14/179) | 7.88 (13/165) | 2.44 (1/41) | -2.44  (-7.22, 2.34,) | -7.82***  (-11.77, -3.88) | -0.06  (-5.76, 5.65) | 5.44  (-3.18, 14.06) |
| (7) Improper dosage prescribed | NA | 40.00 (66/165) | 26.83 (11/41) | -26.83***  (-40.56, -13.10) | NA | NA | 13.17  (-3.36, 29.70) |

**Note:** a. We did not consider Chinese patent medicine in measuring the outcomes in this table.

*p*>0.05, * *p*≤0.05, ** *p*<0.01, *** *p*<0.001

# Appendix Table S4 Prescriptions in the Treatment of a Presumptive Asthma (104 doctors)

| Variable | *Know* | *Can* | *Do* | *Know-Can* Gap | *Can-Do* Gap |
| --- | --- | --- | --- | --- | --- |
| Number of encounters | 104 | 104 | 104 | 208 | 208 |
| 1. Encounters with prescriptions (yes = 1) | 53.85 (56/104) | 50.00 (52/104) | 67.31 (70/104) | 3.85  (-9.79, 17.48) | -17.31*  (-30.55, -4.07) |
| 2. Encounters in which prescribed medicines were loose ^a^ (yes = 1) | NA | NA | 5.77 (6/104) | NA | NA |
| Encounters assessed | 56 | 52 | 64 | 108 | 116 |
| 3. Encounters in which only Chinese patent medicines were prescribed (yes = 1) | 7.14 (4/56) | 11.54 (6/52) | 35.94 (23/64) | -4.40  (-15.41, 6.62) | -24.40**  (-39.74, -9.06) |
| 4. Encounters in which only Western medicines were prescribed (yes = 1) | 53.57 (30/56) | 59.62 (31/52) | 23.44 (15/64) | -6.04  (-24.90, 12.81) | 36.18***  (19.39, 52.97) |
| 5. Encounters in which both Chinese patent medicines and Western medicines were prescribed (yes = 1) | 39.29 (22/56) | 28.85 (15/52) | 40.63 (26/64) | 10.44  (-7.53, 28.41) | -11.78  (-29.29, 5.73) |
| 6. Number of medicines prescribed per encounter | 4.48 (2.40) | 3.92 (2.27) | 2.63 (1.28) | 0.56  (-0.33, 1.44) | 1.30***  (0.64, 1.95) |
| 7. Number of medicines prescribed via injection per encounter | 3.23 (2.91) | 2.96 (2.68) | NA | 0.27  (-0.79, 1.33) | NA |
| 8. Number of non-injection medicines per encounter | 1.25 (1.24) | 0.96 (1.05) | 2.63 (1.28) | 0.29  (-0.15, 0.72) | -1.66***  (-2.10, -1.23) |
| 9. Number of Chinese patent medicines prescribed per encounter | 0.66 (0.84) | 0.56 (0.78) | 1.22 (0.95) | 0.10  (-0.20, 0.41) | -0.66***  (-0.98, -0.34) |

**Note:** a. When providers provided loose medications (medications taken from original packaging and did not give a written or oral prescription) in the standardized patient (SP) visits, the exact name of these medicines and related information is undeterminable. Six encounters with loose medicines were excluded from the rest of the analysis.

*p*>0.05, * *p*≤0.05, ** *p*<0.01, *** *p*<0.001

# Appendix Table S5 Irrational Use of Medicine in the Treatment of a Presumptive Asthma (104 doctors)

| Variable | *Know* | *Can* | *Do* | Total gap | *Know* Gap | *Know-Can* Gap | *Can-Do* Gap |
| --- | --- | --- | --- | --- | --- | --- | --- |
| Number of encounters assessed ^a^ | 52 | 46 | 41 | 41 | 52 | 98 | 87 |
| (1) Only correct medicines prescribed | 7.69 (4/52) | 4.35 (2/46) | 12.20 (5/41) | 87.80***  (77.66, 97.95) | 92.31***  (84.99, 99.62) | 3.34  (-6.24, 12.93) | -7.85  (-19.31, 3.62) |
| (2) Only unnecessary medicines prescribed | 36.54 (19/52) | 45.65 (21/46) | 75.61 (31/41) | -75.61***  (-88.92, -62.30) | -36.54***  (-49.75, -23.32) | -9.11  (-28.73, 10.50) | -29.96**  (-49.85, -10.07) |
| (3) Both correct and unnecessary medicines prescribed | 55.77 (29/52) | 50.00 (23/46) | 12.20 (5/41) | -12.20*  (-22.34, -2.05) | -55.77***  (-69.40, -42.14) | 5.77  (-14.20, 25.74) | 37.80***  (19.60, 56.01) |
| (4) Only potentially harmful medicines prescribed | 0.00 (0/52) | 0.00 (0/46) | 0.00 (0/41) | -- | -- | -- | -- |
| (5) Both correct and potentially harmful medicines prescribed | 0.00 (0/52) | 0.00 (0/46) | 0.00 (0/41) | -- | -- | -- | -- |
| (6) Unnecessary and potentially harmful medicines prescribed | 0.00 (0/52) | 0.00 (0/46) | 0.00 (0/41) | -- | -- | -- | -- |
| (7) Correct, unnecessary, and potentially harmful medicines prescribed together | 0.00 (0/52) | 0.00 (0/46) | 0.00 (0/41) | -- | -- | -- | -- |
| Encounters with any of the following irrational uses of medicines | 80.77 (42/52) | 89.13 (41/46) | 65.85 (27/41) | -65.85***  (-80.55, -51.16) | -80.77***  (-91.59, -69.95) | -8.36  (-22.70, 5.97) | 23.28**  (6.39, 40.16) |
| (1) Antibiotics prescribed | 75.00 (39/52) | 67.39 (31/46) | 63.41 (26/41) | -63.41***  (-78.34, -48.49) | -75.00***  (-63.12, -86.88) | 7.61  (-10.44, 25.65) | 3.98  (-16.25, 24.20) |
| (2) Injections prescribed | 67.31 (35/52) | 73.91 (34/46) | 0.00 (0/41) | 0.00  (0.00, 0.00) | -67.31***  (-80.18, -54.43) | -6.61  (-24.85, 11.64) | 73.91***  (60.31, 87.51) |
| (3) Polypharmacy (> 5 drugs) | 46.15 (24/52) | 34.78 (16/46) | 0.00 (0/41) | 0.00  (0.00, 0.00) | 46.15***  (-59.84, 3-2.47) | 11.37  (-8.20, 30.94) | 34.78***  (20.03, 49.53) |
| (4) Incompatible medicines prescribed | 9.62 (5/52) | 10.87 (5/46) | 0.00 (0/41) | 0.00  (0.00, 0.00) | -9.62*  (-17.71, -1.52,) | -1.25  (-13.38, 10.88) | 10.87*  (1.23, 20.51) |
| (5) Repetitive medicines prescribed | 5.77 (3/52) | 6.52 (3/46) | 4.88 (2/41) | -4.88  (-11.55, 1.80) | -5.77  (-12.17, 0.63) | -0.75  (-10.36, 8.86) | 1.64  (-8.26, 11.55) |
| (6) Medicines prescribed having adverse interactions | 7.69 (4/52) | 6.52 (3/46) | 2.44 (1/41) | -2.44  (-7.22, 2.34,) | -7.69*  (-15.01, -0.38) | 1.17  (-9.15, 11.49) | 4.08  (-4.79, 12.96) |
| (7) Improper dosage prescribed | NA | 50.00 (23/46) | 26.83 (11/41) | -26.83***  (-40.56, -13.10) | NA | NA | 23.17*  (2.98, 43.36) |

**Note:** a. We did not consider Chinese patent medicine in measuring the outcomes in this table.

*p*>0.05, * *p*≤0.05, ** *p*<0.01, *** *p*<0.001

# Appendix Table S6. Logistic Regression Analysis of Factors that Potentially Affect the Prescribing of Antibiotics in *Know*, *Can*, and *Do*

| Variable | *Know*  Odds ratio  (95% confidence  level) | *Can*  Odds ratio  (95% confidence level) | *Do*  Odds ratio  (95% confidence level) |
| --- | --- | --- | --- |
| Number of observations | 201 | 187 | 64 |
| Clinician characteristics |  |  |  |
| Male (yes = 1) | 0.70  (0.37, 1.32) | 0.68  (0.36, 1.29) | 1.44  (0.52, 4.00) |
| Age (year) | 1.02  (0.99, 1.05) | 1.01  (0.98, 1.04) | 1.02  (0.97, 1.07) |
| Junior high school or lower education (yes = 1) | 0.72  (0.22, 2.32) | 0.56  (0.17, 1.84) | 1.50  (0.19, 11.57) |
| Senior high school (yes = 1) | 1.11  (0.60, 2.05) | 1.33  (0.71, 2.52) | 1.11  (0.36, 3.40) |
| Junior college or higher education (yes = 1) | 0.99  (0.51, 1.91) | 0.87  (0.44, 1.73) | 0.77  (0.22, 2.65) |
| (Associate) medical practitioner (yes = 1) | 0.83  (0.29, 2.38) | 0.69  (0.24, 2.00) | 1.52  (0.28, 8.31) |
| Familiarity with the term “clinical pathway” (yes = 1) | 1.12  (0.53, 2.36) | 0.83  (0.40, 1.75) | 0.67  (0.20, 2.27) |
| Received any training on asthma in the previous two years (yes = 1) | -- | -- | -- |
| Received any training on the use of antibiotics in the previous two years (yes = 1) | 1.24  (0.71, 2.19) | 1.19  (0.67, 2.13) | 0.56  (0.20, 1.56) |
| Income from working in clinic (%) | 1.00  (0.98, 1.02) | 1.00.  (0.98, 1.02) | 1.03  (0.95, 1.11) |
| Clinic characteristics |  |  |  |
| Number of village providers (persons) | 0.91  (0.67, 1.24) | 1.02  (0.75, 1.40) | 0.89  (0.52, 1.55) |
| Number of patient visits in the previous month (encounters) | 1.00  (1.00, 1.00) | 1.00  (1.00, 1.00) | 1.00  (1.00, 1.00) |
| Implementation of zero-markup drug policy (yes = 1) | -- | -- | -- |
| Varieties of Western drugs in stock | 1.00  (1.00, 1.00) | 1.00  (1.00, 1.00) | 1.01  (1.00, 1.02) |
| Varieties of Chinese patent medicines in stock | 0.99  (0.99, 1.00) | 1.00  (0.99, 1.00) | 1.01  (1.00, 1.02) |
| Availability of medical instruments/services that may be used for the diagnosis of asthma ^a^ |  |  |  |
| Instruments for chest auscultation (yes = 1) | 1.37  (0.08, 22.36) | 1.23  (0.08, 20.10) | -- |
| Instruments for pulmonary ventilation test (yes = 1) | -- | -- | -- |
| Instruments for bronchodilator test (yes = 1) | -- | -- | -- |
| Instruments for assessment of heart rate, pulse, or blood pressure (yes = 1) | 0.68  (0.06, 7.65) | 0.61  (0.05, 6.87) | -- |
| Instruments for chest X-ray examination (yes = 1) | -- | -- | -- |
| Instruments for blood routine test (yes = 1) | 1.51  (0.50, 4.60) | 1.24  (0.42, 3.66) | -- |
| Instruments for percussion added to chest examination (yes = 1) | -- | -- | -- |
| Availability of intravenous drip (yes = 1) | -- | -- | 1.39  (0.12, 16.48) |
| Availability of intramuscular injection (yes = 1) | 0.45  (0.05, 4.41) | 1.23  (0.08, 20.10) | 1.39  (0.12, 16.48) |
| Keep medical records (yes = 1) | 1.56  (0.75, 3.26) | 1.01  (0.47, 2.20) | 1.16  (0.25, 5.41) |
| Incentives from upper levels tied to prescription assessment (yes = 1) | 1.37  (0.67, 2.81) | 0.88  (0.41, 1.89) | 0.95  (0.26, 3.43) |

**Note:** a. According to standards stated in the Chinese national practice guidelines to treat asthma, seven examinations and/or tests are recommended for the diagnosis of asthma.

# Appendix Table S7. Comparison of Use of Medicines in the Treatment of Presumptive Asthma between Providers Who Gave a Correct or Partially Correct Diagnosis and Those who Gave a Wrong Diagnosis

| Variable | Providers who gave a correct or partially correct diagnosis | Providers who gave a wrong diagnosis | Difference  (95% confidence level) |
| --- | --- | --- | --- |
| Number of encounters | 126 | 221 | 347 |
| 1. Encounters with prescriptions (yes = 1) | 54.76 (69/126) | 59.73 (132/221) | -4.97  (-15.82, 5.89) |
| Encounters assessed | 69 | 132 | 201 |
| 2. Encounters in which only Chinese patent medicines were prescribed (yes = 1) | 1.45 (1/69) | 15.91 (21/132) | -14.46**  (-23.37, -5.55) |
| 3. Encounters in which only Western drugs were prescribed (yes = 1) | 75.36 (52/69) | 34.09 (45/132) | 41.27***  (27.82, 54.72) |
| 4. Encounters in which both Chinese patent medicines and Western medicines were prescribed (yes = 1) | 23.19 (16/69) | 50.00 (66/132) | -26.81***  (-40.70, -12.92) |
| 5. Number of medicines prescribed per encounter | 4.39 (2.63) | 4.06 (2.38) | 0.33  (-0.39, 1.05) |
| 6. Number of medicines prescribed via injection per encounter | 2.61 (2.71) | 2.33 (2.75) | 0.28  (-0.52, 1.07) |
| 7. Number of non-injection medicines per encounter | 1.78 (1.35) | 1.73 (1.37) | 0.06  (-0.34, 0.45) |
| 8. Number of Chinese patent medicines prescribed per encounter | 0.29 (0.57) | 1.00 (0.92) | -0.71***  (-0.95, -0.47) |
| Number of encounters assessed ^a^ | 68 | 111 | 179 |
| (1) Only correct medications prescribed | 14.71 (10/68) | 6.31 (7/111) | 8.40  (-0.41, 17.21) |
| (2) Only unnecessary medicines prescribed | 13.24 (9/68) | 53.15 (59/111) | -39.92***  (-53.43, -26.41) |
| (3) Both correct and unnecessary medicines prescribed | 72.06 (49/68) | 40.54 (45/111) | 31.52***  (17.09, 45.95) |
| Encounters with any of the following irrational uses of medicines | 69.12 (47/68) | 81.98 (91/111) | -12.86*  (-25.48, -0.25) |
| (1) Antibiotics prescribed | 51.47 (35/68) | 72.97 (81/111) | -21.50**  (-35.65, -7.36) |
| (2) Injections prescribed | 55.88 (38/68) | 54.95 (61/111) | 0.93  (-14.16, 16.02) |
| (3) Polypharmacy (> 5 drugs) | 42.65 (29/68) | 35.14 (39/111) | 7.51  (-7.18, 22.20) |
| (4) Incompatible medicines prescribed | 7.35 (5/68) | 11.71 (13/111) | -4.36  (-13.46, 4.75) |
| (5) Repetitive medicines prescribed | 8.82 (6/68) | 8.11 (9/111) | 0.72  (-7.69, 9.13) |
| (6) medicines prescribed having adverse interactions | 14.71 (10/68) | 3.60 (4/111) | 11.10**  (3.12, 19.09) |

**Note:** a. We did not consider Chinese patent medicine in measuring the outcomes below.

*p*>0.05, * *p*≤0.05, ** *p*<0.01, *** *p*<0.001

# Appendix Table S8. Recommended Tests and Examinations that Providers Performed in the Clinical Vignette in the Diagnosis of Presumptive Asthma and the Availability in the Clinics of the Corresponding Medical Instruments

| Instruments for | Requested in *know* | Availability in clinic | Difference  (95% confidence level) |
| --- | --- | --- | --- |
| Chest auscultation (yes = 1) | 14.05 (52/370) | 97.57 (361/370) | -83.51***  (-87.31, -79.72) |
| Pulmonary ventilation test (yes = 1) | 3.24 (12/370) | 0.00 (0/370) | 3.24***  (1.43, 5.06) |
| Bronchodilator test (yes = 1) | 0.00 (0/370) | 0.00 (0/370) | -- |
| Assessment of heart rate, pulse, or blood pressure (yes = 1) | 20.27 (75/370) | 98.65 (365/370) | -78.38***  (-82.59, -74.16) |
| Chest X-ray examination (yes = 1) | 25.41 (94/370) | 0.27 (1/370) | 25.13***  (20.63, 29.64) |
| Routine blood test (yes = 1) | 11.35 (42/370) | 7.84 (29/370) | 3.51  (-0.76, 7.79) |
| Percussion added to chest examination (yes = 1) | 0.00 (0/370) | 100.00 (370/370) | -- |

**Note:** **p <* .05, ***p <* .01, ****p <* .001.

# Appendix Table S9. Irrational Use of Medicine, Without and With Consideration of Chinese Patent Medications

|  | *Know* | | |  | *Can* | | |  | *Do* | | |
| --- | --- | --- | --- | --- | --- | --- | --- | --- | --- | --- | --- |
|  | CPMs Excluded | CPMs Included | Difference  (95% confidence level) |  | CPMs Excluded | CPMs Included | Difference  (95% confidence level) |  | CPMs Excluded | CPMs Included | Difference  (95% confidence level) |
| Number of encounters assessed | 201 | | |  | 187 | | |  | 64 | | |
| Encounters with any of the following irrational use of medicines: | 68.66 (138/201) | 70.65 (142/201) | -1.99  (-11.00, 7.02) |  | 75.40 (141/187) | 81.82 (153/187) | -6.42  (-14.73, 1.89) |  | 42.19 (27/64) | 50.00 (32/64) | -7.81  (-25.17, 9.54) |
| (1) Antibiotics prescribed | 57.71 (116/201) | 57.71 (116/201) | 0.00  (-9.68, 9.68) |  | 55.08 (103/187) | 55.08 (103/187) | 0.00  (-10.11, 10.11) |  | 40.63 (26/64) | 40.63 (26/64) | 0.00  (-17.15, 17.15) |
| (2) Injections prescribed | 49.25 (99/201) | 49.25 (99/201) | 0.00  (-9.80, 9.80) |  | 50.80 (95/187) | 50.80 (95/187) | 0.00  (-10.16, 10.16) |  | 0.00 (0/64) | 0.00 (0/64) | -- |
| (3) Polypharmacy (> 5 drugs) | 33.83 (68/201) | 41.29 (83/201) | -7.46  (-16.93, 2.00) |  | 28.34 (53/187) | 35.83 (67/187) | -7.49  (-16.94, 1.97) |  | 0.00 (0/64) | 7.81 (5/64) | -7.81*  (-14.44, -1.19) |
| (4) Incompatible medicines prescribed | 8.96 (18/201) | 11.44 (23/201) | -2.49  (-8.41, 3.44) |  | 9.09 (17/187) | 12.30 (23/187) | -3.21  (-9.48, 3.06) |  | 0.00 (0/64) | 0.00 (0/64) | -- |
| (5) Repetitive medicines prescribed | 7.46 (15/201) | 10.45 (21/201) | -2.99  (-8.57, 2.60) |  | 5.88 (11/187) | 8.02 (15/187) | -2.14  (-7.30, 3.03) |  | 3.13 (2/64) | 4.69 (3/64) | -1.56  (-8.32, 5.20) |
| (6) Medicines prescribed having adverse interactions | 6.97 (14/201) | 6.97 (14/201) | 0.00  (-4.99, 4.99) |  | 6.95 (13/187) | 7.49 (14/187) | -0.53  (-5.79, 4.73) |  | 1.56 (1/64) | 1.56 (1/64) | 0.00  (-4.33, 4.33) |
| (7) Improper dosage prescribed | NA | NA | NA |  | 35.29 (66/187) | 48.66 (91/187) | -13.37**  (-23.31, -3.43) |  | 17.19 (11/64) | 28.13 (18/64) | -10.94  (-25.43, 3.56) |

**Note:** **p <* .05, ***p <* .01, ****p <* .001.

# Appendix Table S10. Recommended Examination Items in Vignette and SP Visits

| Recommended Examination Item | Predetermined Answer | Prohibited Invasive Examination in SP Visits | Possible Inconsistencies between Vignette and SP Visits | Vignette  % (n/N) | SP Visits  % (n/N) |
| --- | --- | --- | --- | --- | --- |
| Chest/back auscultation* | Exhaled gasping that can be heard in both lungs | No | Yes | 8.65 (9/104) | 19.23 (20/104) |
| Pulmonary ventilation test* | Normal | No | No | 2.88 (3/104) | 0.00 (0/104) |
| Bronchodilator test* | Positive | Yes | Yes | 0.00 (0/104) | 0.00 (0/104) |
| Assessment of heart rate, pulse, or blood pressure* | Normal | No | No | 16.35 (17/104) | 22.12 (23/104) |
| Chest X-ray | Normal | Yes | No | 20.19 (21/104) | 0.00 (0/104) |
| Blood routine | Eosinophils increased, other results are normal | Yes | No | 6.73 (7/104) | 0.00 (0/104) |
| Percussion added to chest auscultation | Voiceless in both lungs | No | Yes | 0.00 (0/104) | 0.00 (0/104) |

**Note:** * indicates it is essential examination for the diagnosis. Otherwise, it is only recommended examination for the diagnosis.
